# Facemask against viral respiratory infections among Hajj pilgrims: A challenging cluster-randomized trial

**DOI:** 10.1371/journal.pone.0240287

**Published:** 2020-10-13

**Authors:** Mohammad Alfelali, Elizabeth A. Haworth, Osamah Barasheed, Al-Mamoon Badahdah, Hamid Bokhary, Mohamed Tashani, Mohammad I. Azeem, Jen Kok, Janette Taylor, Elizabeth H. Barnes, Haitham El Bashir, Gulam Khandaker, Edward C. Holmes, Dominic E. Dwyer, Leon G. Heron, Godwin J. Wilson, Robert Booy, Harunor Rashid, on behalf of the Hajj Research Team

**Affiliations:** 1 National Centre for Immunisation Research and Surveillance, The Children’s Hospital at Westmead and The University of Sydney, Sydney, New South Wales, Australia; 2 Department of Family and Community Medicine, Faculty of Medicine in Rabigh, King Abdulaziz University, Jeddah, Saudi Arabia; 3 Menzies Institute for Medical Research Tasmania, Hobart, Tasmania, Australia; 4 Research Center, King Abdullah Medical City, Mecca, Saudi Arabia; 5 Marie Bashir Institute for Infectious Diseases and Biosecurity, School of Life & Environmental Sciences and School of Medical Sciences, The University of Sydney, Sydney, New South Wales, Australia; 6 Umm Al-Qura University, Mecca, Saudi Arabia; 7 Discipline of Child and Adolescent Health, The Children’s Hospital at Westmead Clinical School, Sydney Medical School, The University of Sydney, Sydney, New South Wales, Australia; 8 Faculty of Medicine, The University of Tripoli, Ain Zara, Tripoli, Libya; 9 NSW Health Pathology - Institute for Clinical Pathology and Medical Research, Westmead Hospital and The University of Sydney, Sydney, New South Wales, Australia; 10 NHMRC Clinical Trials Centre, Faculty of Medicine and Health, The University of Sydney, Sydney, New South Wales, Australia; 11 Rehabilitation Department, Al Jalila Children Specialty Hospital, Dubai, United Arab Emirates; 12 Central Queensland Public Health Unit, Central Queensland Hospital and Health Service, Rockhampton, Queensland, Australia; 13 Department of Laboratory Medicine, Hamad Medical Corporation, Doha, Qatar; Mahidol-Oxford Tropical Medicine Research Unit, THAILAND

## Abstract

**Background:**

In this large-scale cluster-randomized controlled trial (cRCT) we sought to assess the effectiveness of facemasks against viral respiratory infections.

**Methods and results:**

Over three consecutive Hajj seasons (2013, 2014, 2015) pilgrims’ tents in Makkah were allocated to ‘facemask’ or ‘no facemask’ group. Fifty facemasks were offered to participants in intervention tents, to be worn over four days, and none were offered to participants in control tents. All participants recorded facemask use and respiratory symptoms in health diaries. Nasal swabs were collected from the symptomatic for virus detection by reverse transcription polymerase chain reaction. Clinical symptoms and laboratory results were analyzed by ‘intention- to-treat’ and ‘per-protocol’. A total of 7687 adult participants from 318 tents were randomized: 3864 from 149 tents to the intervention group, and 3823 from 169 tents to the control group. Participants were aged 18 to 95 (median 34, mean 37) years, with a male to female ratio of 1:1.2. Overall, respiratory viruses were detected in 277 of 650 (43%) nasal/pharyngeal swabs collected from symptomatic pilgrims. Common viruses were rhinovirus (35.1%), influenza (4.5%) and parainfluenza (1.7%). In the intervention arm, respectively 954 (24.7%) and 1842 (47.7%) participants used facemasks daily and intermittently, while in the control arm, respectively 546 (14.3%) and 1334 (34.9%) used facemasks daily and intermittently. By intention-to-treat analysis, facemask use did not seem to be effective against laboratory-confirmed viral respiratory infections (odds ratio [OR], 1.4; 95% confidence interval [CI], 0.9 to 2.1, p = 0.18) nor against clinical respiratory infection (OR, 1.1; 95% CI, 0.9 to 1.4, p = 0.40). Similarly, in a per-protocol analysis, facemask use did not seem to be effective against laboratory-confirmed viral respiratory infections (OR 1.2, 95% CI 0.9–1.7, p = 0.26) nor against clinical respiratory infection (OR 1.3, 95% CI 1.0–1.8, p = 0.06).

**Conclusion:**

This trial was unable to provide conclusive evidence on facemask efficacy against viral respiratory infections most likely due to poor adherence to protocol.

## Introduction

Viral respiratory infections are a major public health burden, causing serious disease especially in vulnerable populations. Influenza-associated lower respiratory tract disease alone causes over 54 million infections per year, eight million cases of severe illness, and 145,000 deaths across all age groups [1]. Ever-increasing and faster international travel intensifies the transmission of respiratory infections, especially in the setting of mass gatherings such as Hajj pilgrimage in Makkah [2]. The rites of Hajj are performed over five or six days, beginning on the eighth day and ending on the thirteenth day of the last month of the Islamic calendar. Coming from over 180 countries pilgrims converge on Makkah to join a procession of two to three million people who perform a series of physically demanding rituals. Such religious and other mass gatherings amplify the transmission of respiratory viruses by up to eight times [3] and may even accelerate the progression of a pandemic as occurred during the 2009 influenza A(H1N1) pandemic following the Iztapalapa Passion Play mass gathering in Mexico in April 2009 [4]. The current outbreak of coronavirus disease 2019 (COVID-19) is an example of how travel accelerates the spread of respiratory viral infection [5].

Non-pharmacological interventions, such as facemask use, and hand washing have been used to complement pharmacological measures in the prevention and control of viral respiratory infections at mass gatherings with no documented efficacy [6]. There is clinical and experimental evidence that surgical masks and respirators reduce transmission of drug-resistant tuberculosis and influenza from infected patients [7, 8], but randomized trials examining the effectiveness of facemasks against viral respiratory infections in household, community or healthcare settings have been either conflicting or inconclusive [9–15], though at least one randomized controlled trial has suggested protection against influenza by facemasks and hand washing, if applied early after exposure [13].

Inadequate sample size is thought to be an important cause of this discrepancy [16–18]. Therefore we designed a large cluster-randomized controlled trial (cRCT) over three years among pilgrims at Hajj to evaluate the efficacy of facemasks against laboratory-confirmed viral respiratory infections. The rationale of the cluster design was to increase administrative efficiency.

## Methods

### Trial design

Our study was an open label cRCT conducted during Hajj in Mina, Greater Makkah, Saudi Arabia among pilgrims from Saudi Arabia, Australia and Qatar over three Hajj seasons, 2013 to 2015. Mina is an uninhabited valley at the outskirts of Makkah and has about 30,000 tents to accommodate pilgrims for up to five days as part of Hajj rituals. Generally, 50 to 150 pilgrims occupy each large tent, allocated by gender and country of origin, but tents with a much smaller number also exist. Pilgrims in each tent sleep close to each other, head-to-head, have meals and perform rites together hence are considered a cluster. A pilot trial was conducted among Australian pilgrims in 2011 to examine the feasibility of such a study and inform power calculations [19]. Following the Consolidated Standards of Reporting Trials (CONSORT) guidelines (S1 Checklist) for cRCTs participants in respective tents were allocated to intervention or control group as per the trial protocol (S2 Appendix) [20, 21]. Our null hypothesis was that facemask use, according to protocol, does not protect from viral respiratory diseases.

Ethical approval for this study was obtained in Saudi Arabia from the Institutional Review Board of King Abdullah Medical City, Makkah, (IRB Ref. No.: 15–205), in Australia from the Hunter New England Human Research Ethics Committee (Reference No: 13/07/17/3.04), and in Qatar from the Joint Institutional Review Board of Hamad Medical Corporation/Weill Cornell Medical College (Ref: 13–00039).

### Procedure

Hajj pilgrims aged ≥18 years from participating countries, staying in allocated tents and able to provide signed informed consent were included. Pilgrims aged <18 years, or ≥18 who had a known contraindication to mask use, had participated in another randomized trial investigating a medical intervention, refused or were unable to sign the consent were excluded.

Agreement was secured from 318 Hajj tour group leaders for 346 tents, occupied by pilgrims from Saudi Arabia, Qatar and Australia to facilitate the study. The randomization unit of this trial was the accommodation tent. We planned to stratify the randomization by country and gender. Although per protocol computer-generated random number allocation by an offsite research coordinator had been planned, this proved impractical in this field study due to poor internet/mobile phone network at the study sites. Because real-time, effective and smooth communication with the offsite research coordinator responsible for random allocation generation was not possible, coin-tossing by an individual who was not a member of the research team (i.e., a fellow pilgrim who was not a participant in the trial, a tour operator or a medical volunteer at Hajj who was not a study team member) was used. As the intervention of wearing a facemask was visible to participants and investigators, the trial could not be blinded; laboratory staff could be, and were blinded to the intervention.

Trained research team members approached adult pilgrims aged 18 years or older in their assigned tents and explained the study in detail on the first day of each Hajj (October 13^th^ 2013, October 2^nd^ 2014 and September 22^nd^ 2015). Individual research team members were assigned about 15 participants from the first day of Hajj. The researchers gave pilgrims an information sheet and answered their queries. Written informed consent was obtained from pilgrims who agreed to participate in the study. As per the study design, no minor, i.e., person under 18 years of age, was recruited in this study. All participants were asked to complete a baseline questionnaire and were provided with a health diary (S1 Appendix) in their preferred language (Arabic or English) which they were to fill out daily during the trial. Each participant was identified with a unique barcode number on their consent form, baseline questionnaire, health diary and any clinical specimens taken. A post-Hajj diary (S1 Appendix) for an additional three days was planned but a negligible return rate prevented this information being included in the analysis.

**Fig 1:**
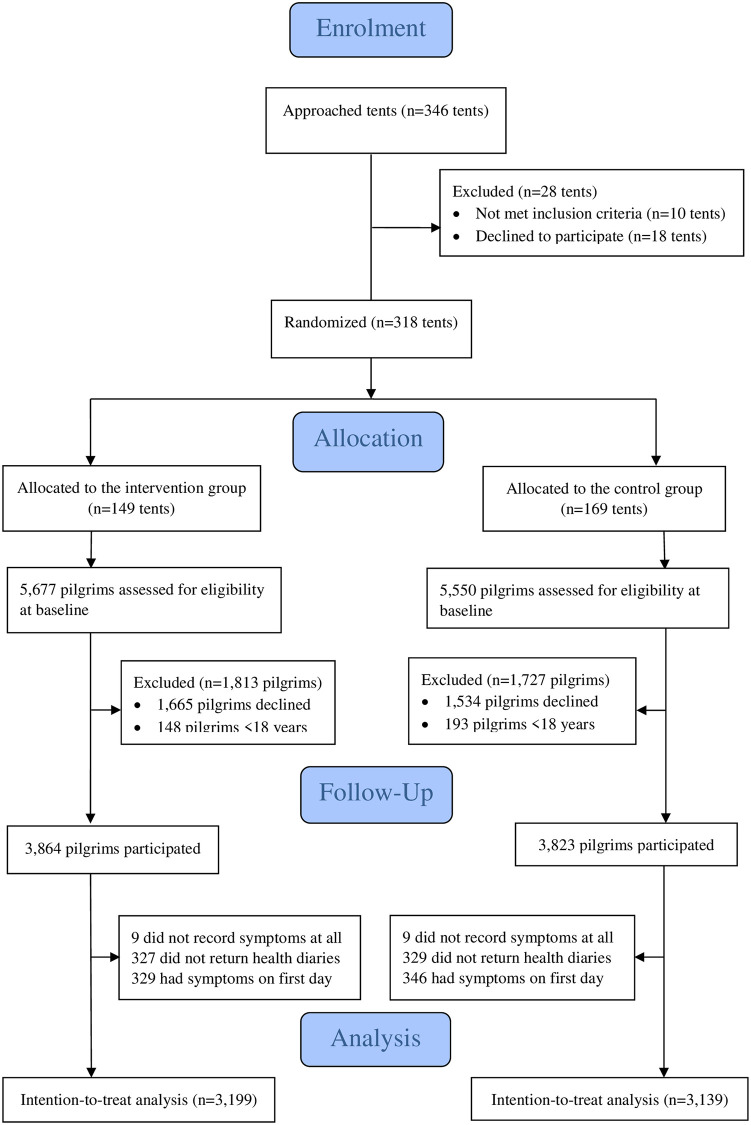
Overall trial flow.

The consent forms and the baseline questionnaires were collected on day one, whereas participants retained diaries for completion over the next four days of Hajj rituals while they were being actively followed (Fig 1).

Each participant in the intervention group was provided with 50 surgical facemasks (3M^™^ Standard Tie-On surgical mask, Cat No: 1816) as well as verbal and printed instructions and demonstration of appropriate facemask usage (S1 Appendix). Pilgrims in the control group were not provided with facemasks and instructions but could use their own masks if they chose to do so. All pilgrims in both study arms were asked to record their facemask usage (including number of masks used and hours worn each study day) in their health diary daily for four consecutive days. Although facemasks were to be worn for 24 hours daily per protocol if possible, for the analysis, pilgrims who used at least one facemask each day during Hajj were considered to have used a facemask during that day, counter to the planned design.

### Measures

As the primary objectives of our trial were to assess the role of facemasks in preventing the acquisition of laboratory-confirmed viral respiratory infections and symptomatic respiratory infection, first primary endpoint was the efficacy of facemasks against laboratory-confirmed viral respiratory infections, and the second primary endpoint was the efficacy of facemasks against clinical respiratory infections in participants.

A total of 464 volunteer researchers were trained by the principal investigators before the study period. Training activities included how to approach pilgrims, the trial processes, explanation and demonstration of facemask use, data collection, follow-up, and sample collection and storage. Study team members were oriented to good clinical practice guidance for the conduct of clinical trials according to the International Council for Harmonisation of Technical Requirements for Pharmaceuticals for Human Use.

The research team visited the study tents twice daily during the study period to ask if the participants developed respiratory or systemic symptoms and collected a nasal swab (FLOQSwabs^™^; COPAN Diagnostics Inc., Murrieta, CA) from those who developed subjective fever together with one respiratory symptom, or two or more respiratory symptoms without fever. Swabs were placed it into UTM^™^ (COPAN) viral transport media. Swabs labelled with the participant’s unique barcode number were stored in an icebox at –20°C before being re-stored by day’s end in a –80°C freezer at the laboratory of the Hajj Research Center at Umm Al-Qura University, Makkah. After Hajj, these swabs were shipped in refrigerated or cold containers to the Centre for Infectious Disease and Microbiology Laboratory Services, Westmead Hospital, NSW, Australia. There, nucleic acid was extracted with the Qiagen bioROBOT EZ instrument (Qiagen, Valencia, CA), and amplification was performed using the Roche LC 480 (Roche Diagnostics GmbH, Mannheim, Germany) instrument. Respiratory viruses were detected using a real-time, multiplex reverse transcription polymerase chain reaction assay targeting human coronaviruses (OC43, 229E and NL63), influenza A and B viruses, respiratory syncytial virus (RSV), parainfluenza viruses 1–3, human metapneumovirus, rhinovirus, enterovirus and adenovirus as described elsewhere [22, 23]. Middle East respiratory syndrome coronavirus (MERS-CoV) assay targeting the upstream region of the E gene (upE) was also performed as described previously [24].

Symptomatic pilgrims were given generic medications for fever and pain, usually acetaminophen.

### Statistical analysis and power calculation

Data from baseline questionnaires and health diaries were entered by trained research staff into customized web-based forms (WUFOO^®^, https://www.wufoo.com), and extracted into Excel sheets. Data checking against paper records was undertaken by four dedicated researchers (M.A., O.B., A.-M.B., M.T).

Statistical analysis was performed using the SPSS Statistics^®^ v25 (IBM, Chicago, IL, USA) and checked by a statistician using SAS V.9.3.

Assuming that the prevalence of symptomatic viral respiratory infections is 30% in controls and the prevalence of laboratory-confirmed viral respiratory infections in controls is approximately 12%, the intervention was considered clinically worthwhile if it reduced the prevalence of clinical respiratory infection or laboratory-confirmed viral respiratory infections by 50%.

Assuming a moderate intra-cluster correlation of 0.1 and a mean of 75 participants per cluster (tent), and inflating the sample by a design effect of 8.4 to account for clustering [25], the sample size required for a cRCT to detect a reduction from 12% to 6% with 80% power at 5% significance is 2976 per arm. An additional inflation factor of 1.2 was included to allow for up to 15% loss to follow-up or incomplete outcome data. This resulted in a sample size of approximately 3500 participants per arm, making a total of 7000.

A descriptive analysis compared the characteristics of participants in the two arms (intervention and control), both at tent level and at individual participant level, as appropriate. Categorical variables were described using frequencies and percentages, and were compared, where appropriate, by using the Chi-squared test. Continuous data were described using the mean and standard deviation, and were compared by the Student’s t-test. The number or proportion of participants with missing data were reported for all variables, but comparisons between groups only included known values, except where otherwise specified. P values and 95% confidence intervals (CIs) were presented without adjustment.

The first and second primary endpoints were analyzed by intention-to-treat analysis according to the participants’ randomized treatment group regardless of treatment actually received. Outcomes were analyzed using a generalized estimating equation statistical model that accounted for the binary distribution of the data and the correlation between participants in the same tent, assuming an exchangeable correlation structure.

Exploratory multivariable analyses examined the effect of randomized treatment on outcomes in models adjusted for demographic factors, facemask usage and compliance with treatment. Subgroup analyses were conducted to compare the effect of treatment between groups of participants: male vs. female, those with known risk factors vs. those without risk factors or risk status unknown for viral respiratory infections, vaccinated against influenza vs. unvaccinated (or vaccination status reported as unknown), smoker vs. non-smoker, compliance with daily facemask use vs. non-compliance, and by pilgrim’s country of origin.

Subgroup analyses used the same statistical model as the primary outcomes and included an interaction term between randomized treatment and subgroup. If the p value for the interaction term was < 0.05, the effect of treatment was described separately within each subgroup. Primary analysis included all participants who reported symptoms at any time during the study period. Eighteen participants who failed to report symptoms were excluded, but those (n = 675) who reported symptoms only at baseline (i.e., on day one) were included. A per-protocol analysis was undertaken amongst participants who were compliant with instructions and reported symptoms daily after the baseline time point. An analysis was performed on participants who were symptom-free at baseline and who completed symptom reports at least once after baseline (i.e., on day one).

For the primary analysis, pilgrims who did not report symptoms daily were assumed to have no change in their symptoms compared to the most recent reporting day. Those who reported no symptom on any study day were assumed to have never developed symptoms while those who reported symptoms on any day were considered symptomatic.

## Results

### Participants

A total of 318 tents that housed 11,227 pilgrims were recruited in both study arms on the first day of each Hajj over three years (13^th^ October in 2013, 2^nd^ October in 2014 and 22^nd^ September in 2015) and followed for the next four days. The number of occupants in each tent varied according to the size of the tent, ranging from 6 to 150 pilgrims per tent. The total number of participants across all study years was 7,687 with an average participation rate of 68.5% (7687 of 11,227) (ranged from 10 to 100%). Of the total 7687 participants, 3864 from 149 tents were assigned to the intervention and 3,823 from 169 tents were assigned to the control group, with an overall participation rate of respectively 68% (3864 of 5686) and 69% (3823 of 5541). Their age ranged from 18 to 95 years (median, 34; mean, 37; standard deviation, 12.3 years), with 53.9% female. Of all participants, 6998 (91%) were from Saudi Arabia and Qatar, and the rest were from Australia. A large proportion of pilgrims 57.6% (4428 of 7687) were recruited in the third year, 2015. The baseline characteristics are shown in Table 1.

**Table 1:** Baseline characteristics of participants and their compliance with facemask and hand hygiene across the study arms.

	Intervention n (%)	Control n (%)
**Tents**		
Total number of recruited tents	149	169
Gulf	137 (91.9)	151 (89.3)
Australia	12 (8.1)	18 (10.7)
2013	26 (17.4)	22 (13)
2014	46 (30.9)	55 (32.5)
2015	77 (51.7)	92 (54.4)
Male tent	71 (47.7)	72 (42.6)
**Participants**		
Total number of participants	3,864	3,823
Gulf countries	3,575 (92.5)	3,423 (89.5)
Australia	289 (7.5)	400 (10.5)
2013	551 (14.3)	474 (12.4)
2014	1,106 (28.6)	1,128 (29.5)
2015	2,207 (57.1)	2,221 (58.1)
Male	1,891 (48.9)	1,651 (43.2)
Mean (standard deviation) age	36.9 (12.1)	37.2 (12.5)
Median (range) age	34 (18–95)	35 (18–95)
With any risk factor	741 (19.2)	715 (18.7)
Smoking as the single risk factor	401 (10.4)	355 (9.3)
Pregnant among women	32 (1.6)	31 (1.4)
Influenza vaccine uptake	1,929 (49.9)	1,887 (49.4)
Facemask use before recruitment	1,057 (27.4)	924 (24.2)
Daily use of facemask	954 (24.7)	545 (14.3)
Intermittent usage	1,842 (47.7)	1,333 (34.9)
Did not use facemask	808 (20.9)	1,672 (43.7)
Used antiseptic/hand rub	1,818 (47)	1,720 (45)
Washed hands frequently (>2 times a day)	2670 (69.1)	2520 (65.9)
Rarely washed hands (1–2 times a day)	578 (15)	619 (16.2)
Never washed their hands	140 (3.6)	178 (4.5)

Overall facemask use was low, even in the intervention tents, with only 24.7% of participants using facemasks daily. Conversely, in the control tents 14.3% participants used facemasks daily. More participants in the intervention group had used a facemask anytime in the weeks before the actual Hajj compared to those assigned in the control group (27.4% vs. 24.2%, p < 0.01).

Slightly more pilgrims in the intervention group than in the control group reported frequent hand washing during Hajj, including their ritual ablutions (69.1% vs. 65.9%, p < 0.01).

**Fig 2:**
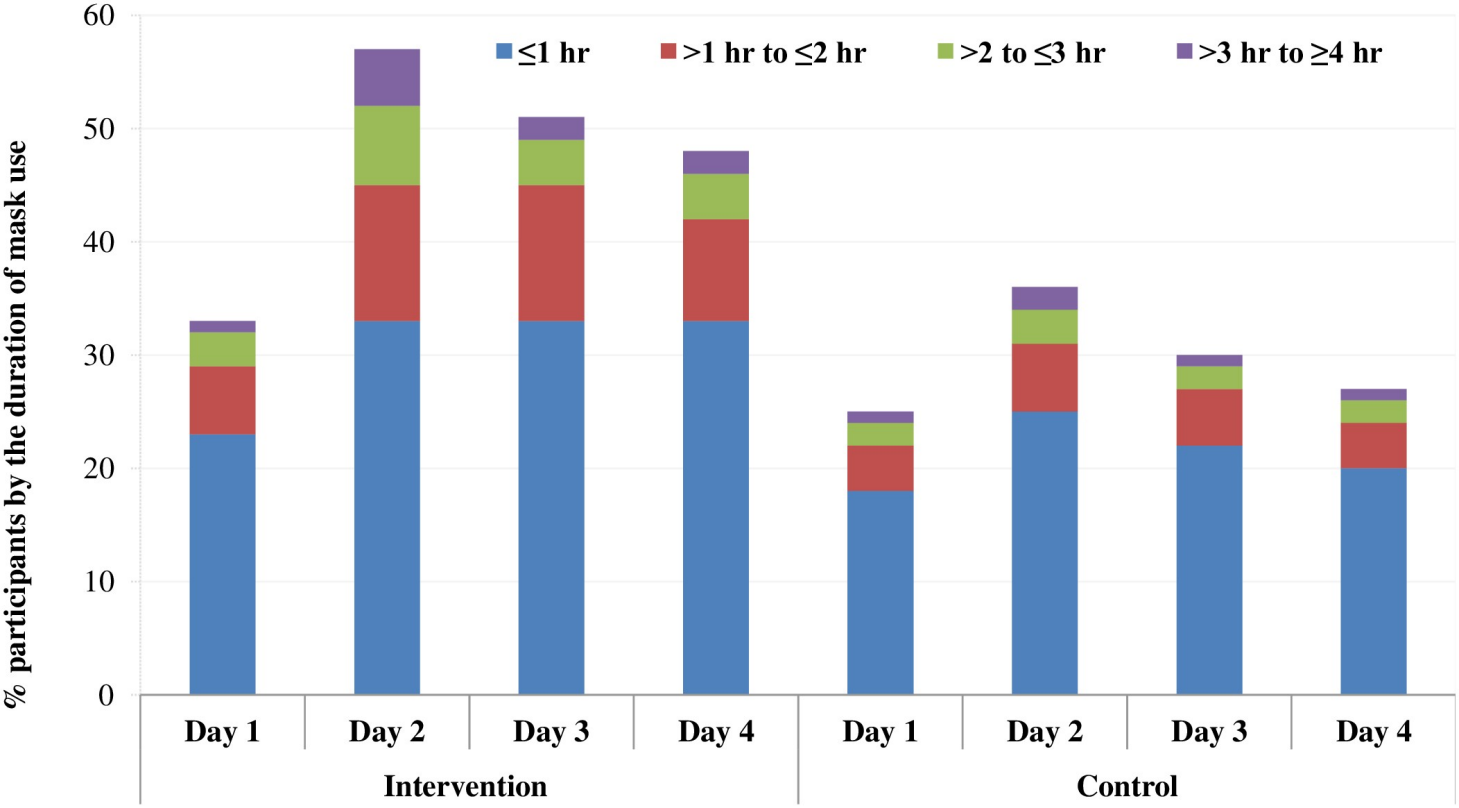
Proportion of facemask using participants by the duration of mask use across the study arms. (This stacked bar chart shows the proportion by subgroup of recorded time of daily facemask use in the intervention group was consistently greater than in the control group).

The proportion of pilgrims who participated in the study and used facemasks ranged between 0 and 50% per tent and the proportion of pilgrims who reported developing clinical respiratory infection in each tent ranged between 0 and 46%. However, in the intervention group, the number by subgroup of recorded time of daily facemask use for at least 4 hours was consistently greater than in the control group (Fig 2).

**Fig 3:**
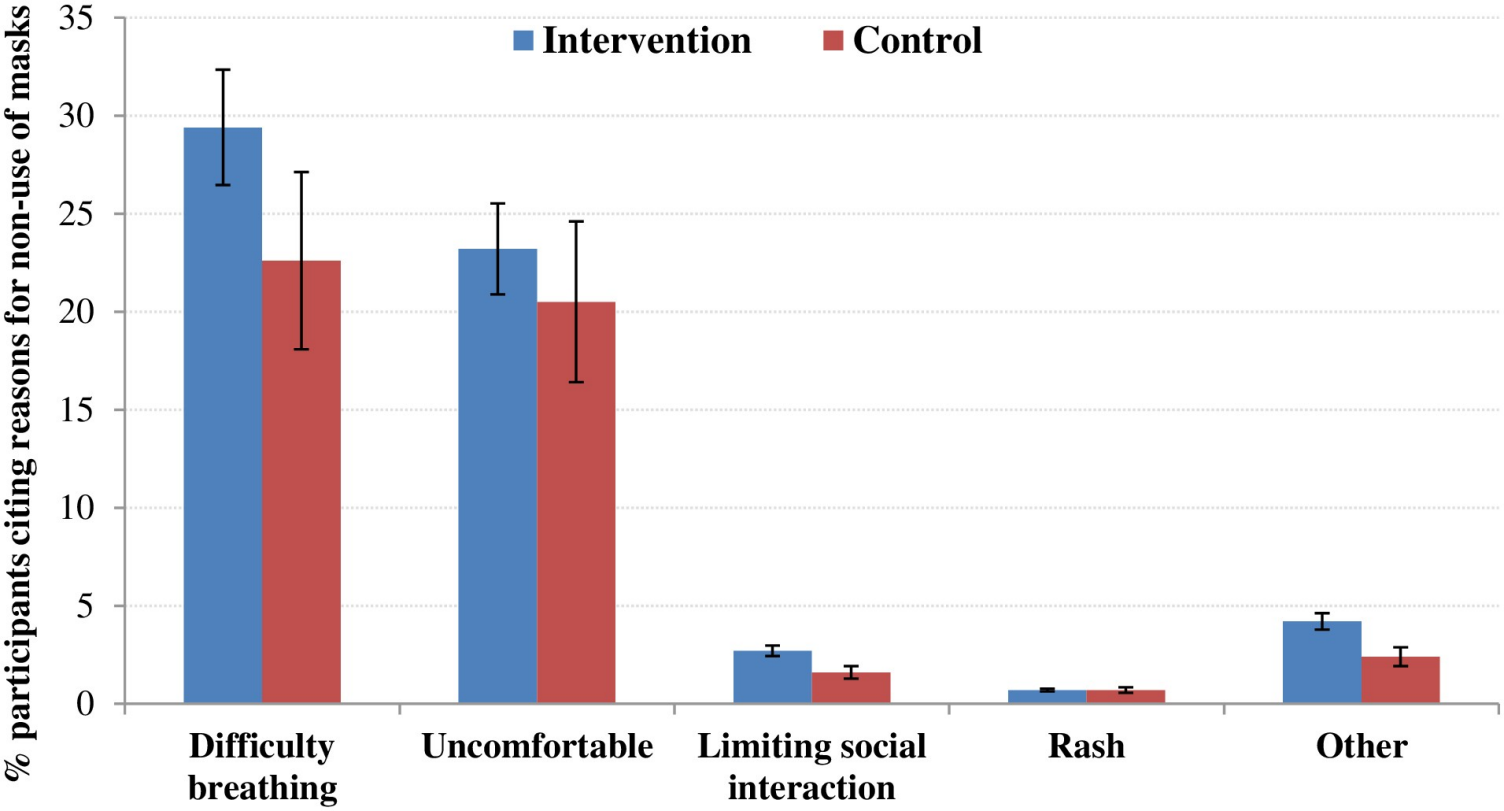
Reasons for not using facemasks across the study arms.

The most common side effects of using facemask were difficulty in breathing (26.2%) and discomfort (22%); a small minority (3%) reported feeling hot, sweating, a bad smell or blurred vision with eyeglasses (Fig 3).

A total of 650 nasal swabs were collected from symptomatic pilgrims in both groups. Overall, one or more respiratory viruses were detected in 277 (42.6%) of samples tested. The most prevalent viruses were rhinovirus (35.1%), influenza, including A/H1N1 and A/H3N2 (4.5%), and parainfluenza (1.7%), and 2% had dual infections (Table 2).

**Table 2:** The attack rate of viral respiratory infections during Hajj in both arms.

	Total	Intervention	Control
	N = 650	N = 358	N = 292
	n (%)	n (%)	n (%)
**Positive**	277 (42.6)	149 (41.6)	128 (43.8)
Rhinoviruses	228 (35.1)	121 (33.8)	107 (36.6)
Influenza A	29 (4.5)	17 (4.7)	12 (4.1)
Influenza A/H1N1	9 (1.4)	5 (1.4)	4 (1.4)
Influenza A/H3N2	14 (2.2)	9 (2.5)	5 (1.7)
Influenza (subtype undetermined)	6 (0.9)	3 (0.8)	3 (1)
Influenza B	1 (0.2)	0 (0)	1 (0.3)
Enteroviruses	10 (1.5)	5 (1.4)	5 (1.7)
Parainfluenza virus 1	5 (0.8)	3 (0.8)	2 (0.7)
Parainfluenza virus 2	1 (0.2)	0 (0)	1 (0.3)
Parainfluenza virus 3	5 (0.8)	3 (0.8)	2 (0.7)
hMPV	2 (0.3)	2 (0.6)	0 (0)
Human coronaviruses	3 (0.5)	2 (0.6)	1 (0.3)
Adenoviruses	3 (0.5)	1 (0.3)	2 (0.7)
RSV	2 (0.3)	2 (0.6)	0 (0)
MERS-CoV	0 (0)	0 (0)	0 (0)
Dual infection	13 (2)	8 (2.2)	5 (1.7)

hMPV: Human metapneumovirus.

In the intention-to-treat analysis, allocation to facemask use was not associated with reduced laboratory-confirmed viral respiratory infections (odds ratio [OR], 1.4; 95% CI, 0.9 to 2.1; p = 0.18) or clinical respiratory infection (OR, 1.1; 95% CI 0.9 to 1.4; p = 0.40) (Table 3).

**Table 3:** Primary and subgroup analyses by intention to treat.

	Intervention n/N (%)	Control n/N (%)	OR (95% CI)	Interaction p value
**Clinical respiratory infection**	354/3,199 (11.1)	322/3,139 (10.3)	1.1 (0.9–1.4)	
Influenza vaccinated	160/1,677 (9.5)	181/1,631 (11.1)	0.8 (0.7–1.1)	**< 0.01**
Not vaccinated or vaccination status reported as unknown	176/1,361 (12.9)	131/1,381 (9.5)	1.4 (1.0–2.0)	
At increased risk	86/621 (13.8)	69/615 (11.2)	1.3 (0.9–1.8)	0.28
Not at increased risk	254/2,439 (10.4)	239/2,399 (10)	1.1 (0.8–1.4)	
Smoking as the single risk factor	40/350 (11.4)	30/311 (9.6)	1.2 (0.7–2)	0.86
Non-smoking	303/2,721 (11.1)	279/2,725 (10.2)	1.1 (0.8–1.4)	
Male	148/1,576 (9.4)	120/1,354 (8.9)	1.1 (0.8–1.5)	0.73
Female	206/1,623 (12.7)	202/1,785 (11.3)	1.2 (0.9–1.6)	
Gulf	332/3,043 (10.9)	287/2,954 (9.7)	1.2 (0.9–1.5)	0.13
Australia	22/156 (14.1)	35/185 (18.9)	0.7 (0.4–1.3)	
**Laboratory-confirmed viral respiratory infection**	96/218 (44)	60/161 (37.3)	1.4 (0.9–2.1)	**-**
Influenza vaccinated	44/106 (41.5)	37/102 (36.3)	1.3 (0.7–2.2)	0.85
Not vaccinated or vaccination status reported as unknown	54/95 (56.8)	21/53 (39.6)	1.3 (0.7–2.2)	
At increased risk	20/42 (47.6)	12/31 (38.7)	3 (1.9–4.9)	0.71
Not at increased risk	71/164 (43.3)	45/114 (39.5)	1.2 (0.7–2.1)	
Smokers	11/28 (39.3)	5/9 (55.6)	0.5 (0.1–2.8)	0.33
Not smokers	80/179 (44.7)	53/138 (38.4)	1.3 (0.8–2)	
Male	45/101 (44.6)	28/50 (56)	0.7 (0.3–1.4)	**0.02**
Female	51/117 (43.6)	32/111 (28.8)	1.9 (1.2–3)	
Gulf countries	15/35 (42.9)	17/48 (35.4)	1.4 (0.6–3.4)	0.96
Australia	81/183 (44.3)	43/113/ (38.1)	1.3 (0.8–2.2)	

In a per-protocol analysis (including only participants allocated to the intervention group who used facemasks daily, and participants allocated to the control group who never used any facemasks) there was no benefit of facemask in preventing laboratory-confirmed viral respiratory infections (OR, 1.2; 95% CI 0.9 to 1.7; p = 0.26) or clinical respiratory infection (OR, 1.3; 95% CI 1.0 to 1.8; p = 0.06) (Table 4).

**Table 4:** Per-protocol analysis: Effect of facemasks against clinical and laboratory-confirmed viral respiratory infections.

Respiratory infection		Intervention n (%)	Control n (%)	OR^a^ (95% CI; p)
***Clinical respiratory infection***	*Did not use facemask*			1.3^a^ (1.0–1.8; 0.06)
	Symptoms present	55 (8)	141 (9)	
	Symptoms absent	648 (92)	1356 (91)	
	*Used facemask*			
	Symptoms present	97 (12)	38 (8)	
	Symptoms absent	731 (88)	425 (92)	
***Laboratory-confirmed viral respiratory infections based on facemask use***	*Did not use facemask*			1.2^b^ (0.9–1.7; 0.26)
	Positive	29 (45)	50 (41)	
	Negative	35 (55)	72 (59)	
	*Used facemask daily*			
	Positive	46 (50)	20 (53)	
	Negative	47 (50)	18 (47)	

^a^Analysis includes only participants from intervention group who used facemasks daily (n = 828) and those from control group who did not use facemasks (n = 1497).

^b^Analysis includes only participants from intervention group who used facemasks daily (n = 93) and those from control group who did not use facemasks (n = 122).

## Discussion

This randomized trial, like other smaller trials [9–15], failed to provide conclusive evidence on facemask efficacy against laboratory-confirmed or clinical respiratory infections. Inconclusiveness of this and previous studies might be attributed in part to respiratory pathogens having multiple routes of transmission including contact with contaminated surface [26, 27] and fecal-oral transmission of some respiratory viruses [28].

The large sample size in our cRCT enabled the comparison of a much larger number of clinical infections (intervention: control = 354: 322) and many more laboratory-confirmed infections (intervention: control = 96: 60) with higher power than the other randomized trials combined. Although unvaccinated pilgrims in the intervention group had a higher rate of clinical respiratory infection than their counterpart in the control group (13% vs. 10%, p = 0.03), this difference is unexplained. However, in previous studies, the prevalence of influenza-like illness among Hajj pilgrims was inversely proportional to their influenza vaccination uptake [29], and vaccinated Hajj pilgrims had 43% reduction in the probability of proven influenza infection [30]. A meta-analysis of six observational studies has shown influenza vaccine to be significantly protective against laboratory-confirmed influenza (relative risk, 0.56; 95% CI, 0.41 to 0.75) [31].

Females in the intervention group of our cRCT were at higher risk of acquiring laboratory-confirmed viral respiratory infections than in the control group (44% vs. 29%; p < 0.01). The reason is unclear, though one possible explanation is Muslim women prefer a loose face cover to a fitted facemask. Over 70% female pilgrims use a face veil during Hajj: one fifth of them use both face veil and mask [32]. Female pilgrims who used a face cover only occasionally (43.2%) tended to have higher rates of clinical respiratory infection compared to those who used it most of the time (36%) [33]. Although we did not assess face cover use by female pilgrims in our study, given that most pilgrims used facemasks only occasionally, the higher rate of viral respiratory infections among women might be due to intermittent use of face cover or even contamination of their masks [9, 33]. When they become wet, facemasks may even increase the transmission of infection, either through becoming more porous or by allowing passage of virus through the mask which is transmitted when the mask is handled [34].

The detection rate of respiratory viruses (43%) in our trial was higher than that reported in other studies (4 to 15%) [35], possibly due to the active case ascertainment strategy employed, including close follow-up of the symptomatic participants. However, the distribution of the viruses was similar to that in other studies: i.e., predominance of rhinovirus, followed by influenza and parainfluenza virus, these three viruses accounting for 97% (269/277) of viruses detected in our study.

No MERS-CoV was detected among the studied participants. Since the emergence of MERS-CoV in Saudi Arabia in 2013, multiple surveillance studies among >10,000 pilgrims from various countries have been undertaken without identifying a Hajj-related case [36, 37]. Now that there is a grave fear of massive acceleration of COVID-19 spread via large population movements [38], other preventive measures including hand and food hygiene, safe drinking water and physical distancing should be encouraged in addition to facemasks [39, 40]. We note that 0.5% of the symptomatic pilgrims tested positive for seasonal coronavirus indicating coronavirus transmission may occur in this setting and that, without the possibility of cross-protection, it is imperative that the Hajj should be scaled-down until the COVID-19 pandemic ends.

Findings of systematic reviews have been conflicting [17, 18, 39, 41–45], the most recent showing protective effects of masks against respiratory viral infection including pandemic coronaviruses across different populations but with low certainty [39, 41]. An observational study conducted at Hajj over four consecutive years (2014 to 2017) found pilgrims who reported using facemasks had higher likelihood of suffering from influenza-like illness symptoms (adjusted relative risk, 1.42; 95% CI, 1.10 to 1.82) and acquiring rhinovirus infection (adjusted relative risk, 1.30; 95% CI, 1.03 to 1.65) [46]. In an earlier study, 20.7% pilgrims who used a facemask reported fever compared with 15.6% who did not (p < 0.01) [47].

The most important limitation of this RCT was that despite much effort to encourage adherence with our protocol, compliance was limited. On the other hand many pilgrims randomized to the control group used facemasks, contrary to the research protocol. The Saudi Arabian Ministry of Health had issued advice to pilgrims to use facemasks while MERS-CoV was circulating in Saudi Arabia during our study period [48], though this was without definitive advice on how to wear masks and the duration of their use. It would have been unethical to counter the Saudi authority’s official advice on facemask use. Our trial does indicate, however, that as a public health intervention, facemask use is not practicable. Another important limitation of the study is that nasal swabs were not performed on the first day when subjects were enrolled. We depended on the reporting of clinical symptoms from day one and followed up directly for four days but did not validate the asymptomatic state with virological testing i.e., some asymptomatic pilgrims could have been virus positive. Longer follow-up was attempted through post-Hajj surveillance, but the low compliance precluded any meaningful analysis. Pilgrims moving from place to place to accomplish Hajj rites made it difficult for researchers to follow them as well as planned. While our study protocol required a clinical sample to be collected in participants with clinical respiratory infection, sampling was performed in some who did not meet the clinical respiratory infection definition whilst others were not swabbed when symptomatic. Though a cRCT design, complying as far as possible with CONSORT guidance, not all occupants in the selected tents participated. On average 69% of tent occupants participated in the trial, ranging widely from 10 to 100%, with possible dilution of the effect of facemask use. The trial was conducted over three years, with uneven recruitment over the years, and most participants (58%) recruited in the final year (2015). The rate of clinical respiratory infection over the study years was 15.5% in 2013, 8.3% in 2014, and 10.7% in 2015, reflecting the known seasonal variability of respiratory viruses, which may have affected the outcome of our study. About 9% participants in each study arm failed to return their diaries or returned dairies without recording symptoms, and another 9% in each group were excluded for being symptomatic on day one of our trial.

Another limitation is that participating tents varied between the study arms (intervention: control = 149: 169) because some tents, although designated as separate units, were later found to be parts of a larger tent. This was more common when several small tour groups were managed under a large tour operator and communal activities (e.g., meals, congregational prayers, sermons) were combined in one large tent. It is also possible that some of these tents were allocated to both intervention and control arms and may have further contributed to dilution of the magnitude of the effect or cluster contamination.

The failure of post-Hajj reporting was another limitation of our study. However, the median incubation periods of the three most common viruses (rhino, influenza and parainfluenza) are <3 days [49], indicating that the majority of the detected infections were acquired after enrolment. Though dangerous to presume very little symptomatic disease post-Hajj this is one explanation for non-adherence with post-Hajj follow-up.

Our cRCT, which was a field study in real time, was unable to refute our null hypothesis. Lack of facemask efficacy observed in this trial could be attributed to limited facemask use by participants (only 24.7% used daily and 47.7% used intermittently in the intervention group), the substantial proportion of participants in the control group who used facemasks, the inability to follow participants after Hajj or the likely contamination of masks [9, 26]. Though more in the intervention group consistently wore masks for defined periods daily, facemask use by controls further reduced the ability of the study to detect differences in infection rates between the study arms.

Due to lack of blinding pilgrims in control tents reporting even mild illness may have reported symptoms because they knew that they had not received the intervention. Also research team members may have been biased towards swabbing such participants assuming them to be less protected, which could have led to an overestimate of the effect of intervention.

The high rate of facemask use (almost 80%) observed among French Hajj pilgrims during the 2009 influenza pandemic year [50], compared to about 55% in a non-pandemic year [3], and in pilgrims from Southeast Asia (e.g., 73% among Malaysian pilgrims in 2007) seem to be due to cultural differences and heightened awareness during a pandemic [51]. The lower uptake of facemasks among participants in our cRCT is similar to the uptake among Saudi Arabian (35 to 57%) and Australian pilgrims (53 to 57%) observed in previous surveys [31, 32, 52, 53], while uptake as low as 0.02% was also recorded among some international pilgrims [54]. Although 78% in the intervention group of our cRCT used facemasks, only a quarter used them regularly. The most common reasons for non-compliance, difficulty in breathing and feeling of discomfort, found also in previous surveys among Hajj pilgrims [52], limited the use of facemasks in this cRCT. These might be strong and important limitations to effective facemask use against respiratory infections, since, in a mass gathering, close contact setting, around the clock protection would be necessary.

Given the number of people who attended Hajj and the close proximity within and among the living quarters, contact transmission from direct contact and indirect contact with contaminated surfaces may have been important and perhaps another reason why these results should be considered inconclusive. There is also the possibility of exposure and infection during travel and prior to Hajj itself. This RCT demonstrates the difficulties of participants’ adherence to instructions and protocol even with active supervision.

Several previous observational studies at Hajj have failed to show the effectiveness of facemasks in preventing respiratory infections [50–52], possibly due also to poor adherence to instructions, although a recent study showed that changing facemask every four hourly reduced the chance of upper respiratory tract infections among Hajj pilgrims (adjusted OR 0.56; 95% CI 0.34 to 0.92; p = 0.02) [55]. In our cRCT, though pilgrims in both intervention and control groups were close to each other day and night, none wore a mask for 24 hours as advised. This may have been an unrealistic expectation. Mask wearing during the COVID-19 pandemic has highlighted the importance of effective and realistic health messaging.

Additional studies with an even larger sample size and more intense supervision would better test the efficacy of facemasks and the role of other interventions, such as hand hygiene, in a mass gathering setting. These will be especially important to evaluate such interventions during the COVID-19 pandemic.

## Conclusion

This trial failed to provide definitive evidence for the effectiveness of facemasks during the Hajj. This was likely due to poor compliance with facemask use. We report difficulties in implementing a large cRCT, evaluating the effectiveness of facemasks against viral respiratory infections including participants’ poor compliance with the protocol, despite active explanation and support.

## Supporting information

S1 Checklist CONSORT, consolidated standards of reporting trials. (DOC)

S1 Appendix Members of the Hajj research team and study tools. (DOCX)

S2 Appendix Study protocol. (DOC)

## References

[1] GBD 2017 Influenza Collaborators. Mortality, morbidity, and hospitalisations due to influenza lower respiratory tract infections, 2017: an analysis for the Global Burden of Disease Study 2017. Lancet Respir Med. 2019;7(1):69–89. 10.1016/S2213-2600(18)30496-X 30553848 PMC6302221

[2] Memish ZA, Steffen R, White P, Dar O, Azhar EI, Sharma A, et al Mass gatherings medicine: public health issues arising from mass gathering religious and sporting events. Lancet. 2019;393(10185):2073–84. 10.1016/S0140-6736(19)30501-X 31106753 PMC7159069

[3] Benkouiten S, Charrel R, Belhouchat K, Drali T, Salez N, Nougairede A, et al Circulation of respiratory viruses among pilgrims during the 2012 Hajj pilgrimage. Clin Infect Dis. 2013;57(7):992–1000. 10.1093/cid/cit446 23839997 PMC7108031

[4] Zepeda-Lopez HM, Perea-Araujo L, Miliar-García A, Dominguez-López A, Xoconostle-Cázarez B, Lara-Padilla E, et al Inside the outbreak of the 2009 influenza A (H1N1)v virus in Mexico. PLoS One. 2010;5(10):e13256 10.1371/journal.pone.0013256 20949040 PMC2951908

[5] Bajema KL, Oster AM, McGovern OL, Lindstrom S, Stenger MR, Anderson TC, et al Persons Evaluated for 2019 Novel Coronavirus—United States, January 2020. MMWR Morb Mortal Wkly Rep. 2020;69(6):166–70. 10.15585/mmwr.mm6906e1 32053579 PMC7017962

[6] Haworth E, Barasheed O, Memish ZA, Rashid H, Booy R. Prevention of influenza at Hajj: applications for mass gatherings. J R Soc Med. 2013;106(6):215–23. 10.1258/jrsm.2012.120170 23761581 PMC3705423

[7] Dharmadhikari AS, Mphahlele M, Stoltz A, Venter K, Mathebula R, Masotla T, et al Surgical face masks worn by patients with multidrug-resistant tuberculosis: impact on infectivity of air on a hospital ward. Am J Respir Crit Care Med. 2012;185(10):1104–9. 10.1164/rccm.201107-1190OC 22323300 PMC3359891

[8] Johnson DF, Druce JD, Birch C, Grayson ML. A quantitative assessment of the efficacy of surgical and N95 masks to filter influenza virus in patients with acute influenza infection. Clin Infect Dis. 2009;49(2):275–7. 10.1086/600041 19522650

[9] MacIntyre CR, Seale H, Dung TC, Hien NT, Nga PT, Chughtai AA, et al A cluster randomised trial of cloth masks compared with medical masks in healthcare workers. BMJ Open. 2015;5(4):e006577 10.1136/bmjopen-2014-006577 25903751 PMC4420971

[10] Jacobs JL, Ohde S, Takahashi O, Tokuda Y, Omata F, Fukui T. Use of surgical face masks to reduce the incidence of the common cold among health care workers in Japan: a randomized controlled trial. Am J Infect Control. 2009;37(5):417–9. 10.1016/j.ajic.2008.11.002 19216002

[11] Cowling BJ, Fung RO, Cheng CK, Fang VJ, Chan KH, Seto WH, et al Preliminary findings of a randomized trial of non-pharmaceutical interventions to prevent influenza transmission in households. PLoS One. 2008;3(5):0002101. 10.1371/journal.pone.0002101 PMC2364646 18461182

[12] Aiello AE, Murray GF, Perez V, Coulborn RM, Davis BM, Uddin M, et al Mask use, hand hygiene, and seasonal influenza-like illness among young adults: a randomized intervention trial. J Infect Dis. 2010;201(4):491–8. 10.1086/650396 20088690

[13] Suess T, Remschmidt C, Schink SB, Schweiger B, Nitsche A, Schroeder K, et al The role of facemasks and hand hygiene in the prevention of influenza transmission in households: results from a cluster randomised trial; Berlin, Germany, 2009–2011. BMC Infect Dis. 2012;12:26 10.1186/1471-2334-12-26 22280120 PMC3285078

[14] MacIntyre CR, Cauchemez S, Dwyer DE, Seale H, Cheung P, Browne G, et al Face mask use and control of respiratory virus transmission in households. Emerg Infect Dis. 2009;15(2):233–41. 10.3201/eid1502.081167 19193267 PMC2662657

[15] Canini L, Andreoletti L, Ferrari P, D’Angelo R, Blanchon T, Lemaitre M, et al Surgical mask to prevent influenza transmission in households: a cluster randomized trial. PLoS One. 2010;5(11): e13998 10.1371/journal.pone.0013998 21103330 PMC2984432

[16] Rashid H, Booy R, Heron L, Memish ZA, Nguyen-Van-Tam J, Barasheed O, et al Unmasking masks in Makkah: preventing influenza at Hajj: Clin Infect Dis. 2012;54(1):151–3. 10.1093/cid/cir826 22187415

[17] Cowling BJ, Zhou Y, Ip DK, Leung GM, Aiello AE. Face masks to prevent transmission of influenza virus: a systematic review. Epidemiol Infect. 2010;138(4):449–56. 10.1017/S0950268809991658 20092668

[18] Bin-Reza F, Chavarrias VL, Nicoll A, Chamberland ME. The use of masks and respirators to prevent transmission of influenza: a systematic review of the scientific evidence. Influenza Other Respi Viruses. 2012l;6(4):257–67. 10.1111/j.1750-2659.2011.00307.x PMC5779801 22188875

[19] Barasheed O, Almasri N, Badahdah AM, Heron L, Taylor J, McPhee K, et al Pilot Randomised Controlled Trial to Test Effectiveness of Facemasks in Preventing Influenza-like Illness Transmission among Australian Hajj Pilgrims in 2011. Infect Disord Drug Targets. 2014;14(2):110–6. 10.2174/1871526514666141021112855 25336079

[20] Campbell MK, Piaggio G, Elbourne DR, Altman DG. Consort 2010 statement: extension to cluster randomised trials. BMJ. 2012;345:e5661 10.1136/bmj.e5661 22951546

[21] Wang M, Barasheed O, Rashid H, Booy R, El Bashir H, Haworth E, et al A cluster-randomised controlled trial to test the efficacy of facemasks in preventing respiratory viral infection among Hajj pilgrims. J Epidemiol Glob Health. 2015;5(2):181–9. 10.1016/j.jegh.2014.08.002 25922328 PMC7103985

[22] Ratnamohan VM, Taylor J, Zeng F, McPhie K, Blyth CC, Adamson S, et al Pandemic clinical case definitions are non-specific: multiple respiratory viruses circulating in the early phases of the 2009 influenza pandemic in New South Wales, Australia. Virol J. 2014;11:113 10.1186/1743-422X-11-113 24942807 PMC4076060

[23] Gaunt ER, Hardie A, Claas EC, Simmonds P, Templeton KE. Epidemiology and clinical presentations of the four human coronaviruses 229E, HKU1, NL63, and OC43 detected over 3 years using a novel multiplex real-time PCR method. J Clin Microbiol. 2010;48(8):2940–7. 10.1128/JCM.00636-10 20554810 PMC2916580

[24] Corman VM, Olschlager S, Wendtner CM, Drexler JF, Hess M, Drosten C. Performance and clinical validation of the RealStar MERS-CoV Kit for detection of Middle East respiratory syndrome coronavirus RNA. J Clin Virol. 2014;60(2):168–71. 10.1016/j.jcv.2014.03.012 24726679 PMC7106532

[25] Machin DC, M J, Tan SB, Tan SH. Sample size tables for clinical studies. 3rd ed: Wiley-Blackwell; 2009.

[26] Hoang VT, Sow D, Belhouchat K, Dao TL, Ly TDA, Fenollar F, et al Environmental investigation of respiratory pathogens during the Hajj 2016 and 2018. Travel Med Infect Dis. 2020;33:101500 [Epub ahead of print]. 10.1016/j.tmaid.2019.101500 31600567 PMC7110696

[27] Killingley B, Nguyen-Van-Tam J. Routes of influenza transmission. Influenza Other Respir Viruses. 2013;7 Suppl 2:42–51. 10.1111/irv.12080 PMC5909391 24034483

[28] Zhang W, Du RH, Li B, Zheng XS, Yang XL, Hu B, et al Molecular and serological investigation of 2019-nCoV infected patients: implication of multiple shedding routes. Emerg Microbes Infect. 2020;9(1):386–9. 32065057 10.1080/22221751.2020.1729071 PMC7048229

[29] Alfelali M, Barasheed O, Tashani M, Azeem MI, El Bashir H, Memish ZA, et al Changes in the prevalence of influenza-like illness and influenza vaccine uptake among Hajj pilgrims: A 10-year retrospective analysis of data. Vaccine. 2015;33(22):2562–9. 10.1016/j.vaccine.2015.04.006 25887084

[30] Alfelali M, Barasheed O, Koul P, Badahdah AM, Bokhary H, Tashani M, et al Influenza vaccine effectiveness among Hajj pilgrims: a test-negative case-control analysis of data from different Hajj years. Expert Rev Vaccines. 2019;18(10):1103–14. 10.1080/14760584.2019.1646130 31322451

[31] Alqahtani AS, Rashid H, Heywood AE. Vaccinations against respiratory tract infections at Hajj. Clin Microbiol Infect. 2015;21(2):115–27. 10.1016/j.cmi.2014.11.026 25682277

[32] Alqahtani AS, Wiley KE, Tashani M, Willaby HW, Heywood AE, BinDhim NF, et al Exploring barriers to and facilitators of preventive measures against infectious diseases among Australian Hajj pilgrims: cross-sectional studies before and after Hajj. Int J Infect Dis. 2016;47:53–9. 10.1016/j.ijid.2016.02.005 26875699 PMC7110465

[33] Choudhry AJ, Al-Mudaimegh KS, Turkistani AM, Al-Hamdan NA. Hajj-associated acute respiratory infection among hajjis from Riyadh. East Mediterr Health J. 2006;12(3–4):300–9. 17037698

[34] Isaacs D, Britton P, Howard-Jones A, Kesson A, Khatami A, Marais B, et al Do facemasks protect against COVID-19? J Paediatr Child Health. 2020;56(6):976–7. 10.1111/jpc.14936 32542946 PMC7323223

[35] Gautret P, Benkouiten S, Al-Tawfiq JA, Memish ZA. Hajj-associated viral respiratory infections: A systematic review. Travel Med Infect Dis. 2016;14(2):92–109. 10.1016/j.tmaid.2015.12.008 26781223 PMC7110587

[36] Al-Tawfiq JA, Benkouiten S, Memish ZA. A systematic review of emerging respiratory viruses at the Hajj and possible coinfection with Streptococcus pneumoniae. Travel Med Infect Dis. 2018;23:6–13. 10.1016/j.tmaid.2018.04.007 29673810 PMC7110954

[37] Rashid H, Azeem MI, Heron L, Haworth E, Booy R, Memish ZA. Has Hajj-associated Middle East Respiratory Syndrome Coronavirus transmission occurred? The case for effective post-Hajj surveillance for infection. Clin Microbiol Infect. 2014;20(4):273–6. 10.1111/1469-0691.12492 24313466 PMC7128364

[38] Chen S, Yang J, Yang W, Wang C, Bärnighausen T. COVID-19 control in China during mass population movements at New Year. Lancet. 2020;395(10226):764–6. 10.1016/S0140-6736(20)30421-9 32105609 PMC7159085

[39] Chu DK, Akl EA, Duda S, Solo K, Yaacoub S, Schünemann HJ, et al Physical distancing, face masks, and eye protection to prevent person-to-person transmission of SARS-CoV-2 and COVID-19: a systematic review and meta-analysis. Lancet. 2020; 395(10242):1973–1987. 10.1016/S0140-6736(20)31142-9 32497510 PMC7263814

[40] Wilder-Smith A, Freedman DO. Isolation, quarantine, social distancing and community containment: pivotal role for old-style public health measures in the novel coronavirus (2019-nCoV) outbreak. J Travel Med. 2020;27(2). 10.1093/jtm/taaa020 PMC7107565 32052841

[41] Liang M, Gao L, Cheng C, Zhou Q, Uy JP, Heiner K, et al Efficacy of face mask in preventing respiratory virus transmission: A systematic review and meta-analysis. Travel Med Infect Dis. 2020;101751. 10.1016/j.tmaid.2020.101751 [Epub ahead of print]. PMC7253999 32473312

[42] MacIntyre CR, Chughtai AA. A rapid systematic review of the efficacy of face masks and respirators against coronaviruses and other respiratory transmissible viruses for the community, healthcare workers and sick patients. Int J Nurs Stud. 2020; 108: 103629 [Epub ahead of print]. 10.1016/j.ijnurstu.2020.103629 32512240 PMC7191274

[43] Saunders-Hastings P, Crispo JAG, Sikora L, Krewski D. Effectiveness of personal protective measures in reducing pandemic influenza transmission: A systematic review and meta-analysis. Epidemics. 2017;20:1–20. 10.1016/j.epidem.2017.04.003 28487207

[44] Smith SM, Sonego S, Wallen GR, Waterer G, Cheng AC, Thompson P. Use of non-pharmaceutical interventions to reduce the transmission of influenza in adults: A systematic review. Respirology. 2015;20(6):896–903. 10.1111/resp.12541 25873071 PMC4699551

[45] Jefferson T, Del Mar CB, Dooley L, Ferroni E, Al-Ansary LA, Bawazeer GA, et al Physical interventions to interrupt or reduce the spread of respiratory viruses. Cochrane Database Syst Rev. 2011;(7):CD006207 10.1002/14651858.CD006207.pub4 21735402 PMC6993921

[46] Hoang VT, Ali-Salem S, Belhouchat K, Meftah M, Sow D, Dao TL, et al Respiratory tract infections among French Hajj pilgrims from 2014 to 2017. Sci Rep. 2019;9(1):17771 10.1038/s41598-019-54370-0 31780750 PMC6883043

[47] Maslamani YAMC, A. J. Health related experiences among international pilgrims departing through King Abdul Aziz international airport, Jeddah, Saudi Arabia, Hajj 1431 H (201 0). Saudi Epidemiology Bulletin. 2011;18:42–4.

[48] Algarni H, Memish ZA, Assiri AM. Health conditions for travellers to Saudi Arabia for the pilgrimage to Mecca (Hajj) - 2015. J Epidemiol Glob Health. 2016;6(1):7–9. 10.1016/j.jegh.2015.07.001 26184362 PMC7320520

[49] Lessler J, Reich NG, Brookmeyer R, Perl TM, Nelson KE, Cummings DA. Incubation periods of acute respiratory viral infections: a systematic review. Lancet Infect Dis. 2009;9(5):291–300. 10.1016/S1473-3099(09)70069-6 19393959 PMC4327893

[50] Gautret P, Vu Hai V, Sani S, Doutchi M, Parola P, Brouqui P. Protective measures against acute respiratory symptoms in French pilgrims participating in the Hajj of 2009. J Travel Med. 2011;18(1):53–5. 10.1111/j.1708-8305.2010.00480.x 21199143

[51] Deris ZZ, Hasan H, Sulaiman SA, Wahab MS, Naing NN, Othman NH. The prevalence of acute respiratory symptoms and role of protective measures among Malaysian hajj pilgrims. J Travel Med. 2010;17(2):82–8. 10.1111/j.1708-8305.2009.00384.x 20412173

[52] Barasheed O, Alfelali M, Mushta S, Bokhary H, Alshehri J, Attar AA, et al Uptake and effectiveness of facemask against respiratory infections at mass gatherings: a systematic review. Int J Infect Dis. 2016;47:105–11. 10.1016/j.ijid.2016.03.023 27044522 PMC7110449

[53] Alqahtani AS, Althimiri NA, BinDhim NF. Saudi Hajj pilgrims’ preparation and uptake of health preventive measures during Hajj 2017. J Infect Public Health. 2019;12(6):772–6. 10.1016/j.jiph.2019.04.007 31023600

[54] Elachola H, Assiri AM, Memish ZA. Mass gathering-related mask use during 2009 pandemic influenza A (H1N1) and Middle East respiratory syndrome coronavirus. Int J Infect Dis. 2014;20:77–8. 10.1016/j.ijid.2013.12.001 24355682 PMC7119095

[55] Alasmari AK, Edwards PJ, Assiri AM, Behrens RH, Bustinduy AL. Use of facemasks and other personal preventive measures by Hajj pilgrims and their impact on health problems during the Hajj. J Travel Med. 2020; 10.1093/jtm/taaa155 [Epub ahead of print]. 32901805

