# Supplementary material for: Facemask against viral respiratory infections among Hajj pilgrims: A challenging cluster-randomized trial
Source: PLoS One. 2020 Oct 13;15(10):e0240287. doi: 10.1371/journal.pone.0240287 (PMC7553311; doi:10.1371/journal.pone.0240287)
Supplement: S1 Appendix — (DOCX) [file pone.0240287.s002.docx]

**S1 Appendix**

**TABLE OF CONTENTS**

|  | **Page** |
| --- | --- |
| **Members of the Hajj Research team**…………………………………….…. | 2 |
| **Baseline Questionnaire English**....……………………………………….…. | 7 |
| **Instructions to Use Facemasks** **English**…….………………………………. | 10 |
| **Hajj Diary English**………………………………………………….……….. | 11 |
| **Post-Hajj Diary English**……………………………………………….….… | 20 |
| **Baseline Questionnaire Arabic**………………………………………….….. | 25 |
| **Instructions to use facemask Arabic**……………………………………..… | 27 |
| **Hajj Diary Arabic**…………………………………………………………… | 28 |
| **Post-Hajj Diary Arabic**…………………………………...………………… | 37 |
| **Consent form English**…………………………………...……………..…… | 42 |
| **Consent form Arabic**…………………………………...…………………… | 44 |

**Members of the Hajj Research team**

**Australia**: Harunor Rashid, Robert Booy, Elizabeth A. Haworth, Leon G. Heron, Dominic E. Dwyer, Edward C. Holmes, Mohammad I. Azeem, Jen Kok, Janette Taylor, Elizabeth H. Barnes, Gulam Khandaker, Mohamed Tashani.

**Saudi Arabia**: Osamah Barasheed, Mohammad Alfelali, Al-Mamoon Badahdah, Hamid Bokhary, Nedal Almasri, Jassir Alshehri, Ghassan Matbouly, Jamil Samkari, Nadeen Kalantan, Mohammed Alhefzi, Hisham Alqari, Mukhtaar Sayid, Bayan Hariri, Moataz Fakeerah, Daniah Bondagji, Mohammed Alluhidan, Sami Mushta, Saeed Alsharif, Mohammed G Asiri, Rakan Ikram, Ibtihal Malawi, Ebtehal Matar, Atheer Alshareif, Israa Kalantan, Eatimad Alalawi, Afnan AlGhamdi, Amani Koshak, Ameerah Alkhaldi, Inaam Al-Nami, Anwar Howsawi, Bashaier Fairaq, Bushra Maghrabi, Tafaol Murad, Hanan Alzahrani, Kholood Almehmadi, Doaa Milibari, Rehab Hafiz, Rawdhah Kalantan, Shahad Al-Ansari, Aeshah Rajab, Anood Alfahmy, Ghaida Ali, Fatimah Abu naji, Lujin Hassan, Lulwah Althumali, Layla Farhat, Najlaa Baddour, Hibh Alandanusi, Waad Alqurashi, Sumayyah Fallata, Azhar Alharbi, Joud Bahakeem, Abrar Alshareef, Badr Rawa, Ahmed Alghamdi, Ahmed Muqadimi, Osama Alamri, Jehad Qutub, Abdulrahman Al-Ghamdi, Abdurrahman Mirza, Abdulghafur Alandijani, Omar Qoqandi, Faisal Mandourah, Muhammad Alghamdi, Mohammed Mahboob, Mohannad Alsulami, Moayyd Hinnawi, Naif Hawsawi, Nawaf Dhabab, Ahmed Balamash, Mohammed Bawazir, Raif Nassir, Mohammed AlAsmari, Faisal Alzahrani, Abdulrahman Alomari, Ahmad Makeen, Ibraheem Almani, Ahmed Baabdullah, Osama Alamoudi, Ahmed Alzhrani, Ahmed Bagabas, Ahmad Ahmad, Anwar Alammari, Ayman Alghamdi, Badr Alaifan, Badr Al Dahlawi, Turki Almalki, Thamer Zoghbi, Hussam Patwa, Hasan Ghannam, Hussien Alkully, Samir Alsulaimani, Samee Al Heraki, Saad AlGhamdi, Sultan AlBalawi, Sultan Albukhari, Saleh Algamdi, Abdululah Alsolami, Abdullah Alghanmi, Abdulellah Alturkistani, Abdullrhman Alayad, Abdulrahman Althagafi, Abdulrahman Makki, Abdulrahman Khinkar, Abdulaziz Alalawi, Abdulaziz Alhoqail, Abdulaziz Alshoaibi. Abdullah Aldour, Abdulhadi Towairqi, Ali Ali, Ali Alshubaily, Firas Atwah, Majed Daqeeq, Mohammed Aljunaid, Mohammed Alghamdi, Mohammed Alsefri, Mohammed Alamoudi, Mohammed Alghamdi, Mohammad Melibary, Mohammad Bakhaidar, Mohammad Albogami, Mohammed Almoflihi, Muaath AlGhamdi, Mutaz Abdulhaq, Monther Farghali, Mohannad Khyyat, Moayad Banjar, Wael Almaghthawi, Wael Khalifa, Yasser Halabi, Mohanad Aljohani, Riyadh Alharbi, Moayad Sumnudi, Sultan Al Jaid, Rayan Makeen, Mahmoud Eid, Mohammed Alaryni, Abdulrahman Qahtani, Saud Bakhsh, Turki Alkharji, Ahmed Qadah, Albraa Kashegari, Ahmad Alabbasi, Abdulmohsen Al-Sofi, Meshary Alhassni, Nawaf Alharbi, Ahmad Al Ahdal, Abdulghani Alserafi, Ibrahim Alomry, Mohammed Kadi, Abdulrahman Almalki, Bassel Katib, Ibrahim Sameer, Fares Alnajjar, Mohammed Hawsawi, Rayan Mohammad, Ebtihal Turkistani, Abrar Tawakoul, Arwa Bajabaa, Areej Alzaidi, Ashar Almusallam, Asraa Turkistani, Asmaa Alattas, Alshaima Alghamdi, Esraa Kashkari, Elaf Altwairqi, Elaf Alrehaili, Elaf Khalifa, Inas Magharbil, Abrar Salloma, Arwa Alzaidi, Arej Fadel, Afnan AL Gothami, Amal Al-Saedi, Alaa Binsalman, Aya Kutbi, Baraah Tatwany, Basmah Fallata, Bashayer Al Mutairi, Bashaer Alrefaie, Bashayer Al-huthali, Bashayer Alsaati, Bashaer ALzahrani, Bushra Fallatah, Bushra Alattas,

Bushra Alhajjaji, Banan Almalki, Bayan Zamil, Tamador Alghamdi, Tahani Al-Ghamdi, Jenan Jawi, Haneen Sibieh, Kholoud Natto, Duaa Eid, Reem Alamoodi, Sumaia Felimban, Etaf Kassem, Faten Althobaiti, Fadya Althobaiti, Maria AL-Jehani, Muneerah Al-youbi, Nadeen Bugis, Duaa Aiash, Duaa Assaqaf, Duaa Almouallimi, Rania Iraqi, Rasha Qurashi, Rasha Baqis, Raghad Jamal Aldeen, Ruqaiah Baharoon, Ranad Medhir, Renad Gashlan, Renad Aljohani, Randa Al-Bloushy, Raneen Abu Saadah, Rahaf Shafi, Rawan Gaafar, Reem Alshareef, Zahra Othman, Sara Aljuaid, Sara Al-Ghfari, Salma Sait, Samaa Sangouf, Samar Alsubhi, Sahar Alharbi, Samar Al-harbi, Sana’a Kelantan, Shahad Aldor, Shahd Alshareef, Shaimaa Halabi, Shaimaa Hawsawi, Seba AlHarbi, Azzah Azzouz, Alyaa Idris, Fatimah Alosaimi, Fatimah Alsomali, Lujain Al-Thakafi, Lujain Abdalwassie, Lama Alarabi, Lina Alsaiari, Majedah Alshammari, Mahacen Alnadwi, Mada Abdulhaq, Mada Al Zahrani, Maradi Murad, Maram AlShareef, Marwah Hadidi, Nojoud Benhli, Najwa Mohammad, Nada Almuqati, Noor Alessa, Noura Bakhsh, Nuran Sultan, Norah Alotaibi, Heba Waez, Heba Al-Qethami, Heba Alsheikh, Hebah Alwafi, Hoda Al-Sayid, Hadeel Khoj, Wejdan Makeen, Woud AlMusallam, Waed Yaseen, Wafa Sohail, Sara Fallata, Abdel Mejid Mohamed, Abdulkarim Al-Sabyani, Abdullah Elhosiny, Abdullah Alsayed, Abdullah Nawab, Abdullah Alharbi, Abdulraheem Al-Sadat, Abdulrahman Aldarkhbani, Abdulrahman Alnaser, Abdulrahman Allahyani, Abdulrahman Bazaid, Abdulrhman Al-Malki, Abdulrhman Kinsara, Abrar Khalil, Abrar Ainousa, Abrar Ghulam, Afaf Ebraheem Mas, Ahmad Charbatji, Ahmad Altalhi, Ahmad Bimah, Ahmad Maqadmi, Ahmad Albeshri, Ahmed Sindi, Ahmed Aljuhani, Ahmed Almikhlafi, Aisha Muhammed Memon, Alaa Khoja, Alaa Habib, Alhassan Alhasani, Ali Medher, Ali Alkhathami, Ali Al-Attas, Abdullah Alqarni, Amal Faheem, Amal Alsaedi, Amani Hussin, Amir Khogeer, Ammar Alfattni, Ammar Almaghrabi, Ammar Alaaddin, Anas Salman, Anwar Alharbi, Aqeel Alkhiri, Areej Abdul- Gader, Arwa Shaheen, Arwa Alasmari, Asma Mansoor, Asmah Aldobashi, Ataa Mesbah, Awnallah Al-Otaibi, Ayah Istanbouli, Badriah Aldeaiq, Banan Bawazeer, Bandar Ghonaim, Bashayer Alsaadi, Bassam Elkhouly, Bassil Aladani, Bayan Fatani, Bshaer Badakhan, Bushra Al-Harbi, Dania Shaikh, Daniah Alnemari, Deemah Alindonosi, Elaf Tayeb, Elaf Albasheri, Emad Alharbi, Eman Al Hindi, Eman Almarwani, Ensaf Fatani, Eyaad Ghallab, Faisal Alterazi, Faisal Almaabadi, Faisal Mahmood, Ghadah Althbiti, Ghadi Alotaibi, Ghaliah Al-Haqas, Hadeel Alhassani, Hamad Alhilabi, Hamis Alalhareth, Hanadi Al-Thobaiti, Hashem Moafa, Hassan Almalki, Hayaa Zaki, Heba Aziza, Hind Alrefai, Hisham Alkhuzaei, Horia Abou Shousha, Huda Mansoor, Hussain Jammal, Hussam Rawas, Jawaher Alqurashi, Jomana Ajawi, Jumana Melebari, Jumanah Al-Saedi, Khadeejah Aljifri, Khalid Alsubaie, Leenah Abdulgader, Lin Charbatji, Linah Zamzami, Maan Alraddadi, Mahmood Yasawy, Mahmoud Chaker, Majed Alamoudi, Majid Jawa, Malek Alsairafi, Maram Albarakati, Marium Iqbal, Marwa Hawsawi, Masheal Bawahhab, Mohammad Alzhrani, Mohammad Shaheen, Mohammed Algarni, Mohammed Alzahrani,

Mohammed Al-Fageeh, Mohammed Alaamri, Mohammed Al-Jeddawi, Mohammed Alshreef, Mohammed Alsaggaf, Mohammed Dumyati, Mohammed Al- Mikhlafi, Mohsen Alzamanan, Mona Alghamdi, Muhab Hindi, Muteb Almarwani, Nada Telmesani, Nada Mohammed, Naif Alhowaiti, Nasser Alshehri, Nedaa Karami, Neveen Awad, Nizar Almaghrabi, Nojoud Hli, Nuha Jazzar, Omar Alzhrani, Omar Alotaibi, Osama Alharbi, Qutaibah Aldurrah, Raghad Namnqani, Rahma Al-Ghamdi, Raid Alghamdi, Rana Almimoni, Rana Abbas, Raneem Rawa, Rayyan Alqurayyan, Razan Melibari, Reem Altaifi, Reem Bajunaid, Reem Altowairqi, Reem Alenazi, Reem Alghamdi, Refal Aziz Al-Rahman, Reham Bin Hassan, Roaa Khan, Rowaynah Aziz Al-Rahman, Ruba Alshaikh, Saad Algarni, Saadiah Balkhy, Saeed Balubaid, Safa'A Al-Hasani, Sahar Futtiny, Salman Melhem, Salwa Alotaibi, Samah Alqurashi, Samaher Melybari, Sana Nargis, Sara Alshehtha, Sarah Radwan, Saud Bakhsh, Shahad Bamani, Shahad Alharbi, Shahd Hafiz, Shoroug Alkhabiry, Sofana Alqawsi, Sultan Alzahrani, Sultan Shaqra, Thamer Alanazi, Wafa Alkhuzaie, Wafa Sidiqqi, Wafaa Altaezi, Wafaa Alharbi, Walid Almutairi, Wasaif Aljuhany, Wed Jawa, Yaser Badawood, Yasser Hadi, Yousef Alzahrani, Abaad Al-Mutairi, Abdulaziz Ajaj, Abdullah Tai, Abdullah Ashour, Abdullah Aljohni, Abdullah Alshehri, Abdullateef Allebdi, Abdullateef Alzhrani, Abdulmajeed Alzahrani, Abrar Alnami, Adel Almaymuni, Afnan Joudah, Ali Fadel, Ali Alshehri, Ali Alelyani, Ali Alkhulaifi, Amal Alnakhli, Amani Alharbi, Anas Heji, Arwa Badakhan, Asal Arbaeen, Asmaa Nassir, Basim Almutairi, Atrab Bayazeed, Bushra Alahmadi, Dania Almunami. Dareen Alsaidalany, Ebtehal Yamani, Ebtesam Alghamdi, Eman Alharbi, Eman Kotbi, Fahad Altowairqi, Faisal Althobaiti, Faisal Alsobyani, Farraj Al-Zahrani, Ghadah Althbiti, Ghadah Alshehri, Hadeel Alqahtani, Hanan Mughallis, Haneen Taher, Heba Bayoumi, Hesham Essa, Hussein Alshamrani, Khalil Alghamdi, Malak Alshammari, Maram Albarakati, Marwah Bin-Garhom, Marwan Albeshri, Mohamed Bayoumi, Mohammad Althobaiti, Mohammed Toras, Mohammed Al Thebyani, Naif Alzahrani, Nasheal Bawahhab, Noura Al-Zahrani, Ohoud Alharbi, Radwan Badr, Rahma Aljedaani, Rakan Alnefaie, Rawa'A Al- Maghrabi, Rehab Alshamrani, Reham Abdulgader, Riyadh Alharbi, Sara Al-Ghfari, Sarah Aljoudi, Saud Alzhrani, Somayah Alsolami, Suliman Badi, Sultan Alghamdi, Tawfiq Al Laylah, Wasfi Almusaddi, Wijdan Alzhrani.

Qatar: Haitham El-Bashir, Godwin Justus Wilson.

**Baseline Questionnaire English**

Date .…../…..…/…………. Tent no…….……. Intervention Tent/ Control Tent Barcode # |__|__||__|__||__|__|

Cluster-randomised controlled trial to test the effectiveness of facemasks in preventing respiratory virus infection among Hajj pilgrims

First name: …………………………………. Surname: ……..………...……….….… DOB ..…./……/……… (Age:..……….)

Country of residence………………………………Country of birth ………………………………Gender: □ Male; □Female

Ethnicity: …………………..........…….…………..…..…. Occupation: ………………….…………………………………...………

Address in the country of residence:……………………………………………………………………………………………………

…………………………………………………………………………………………….…………………………………………………

Email:………………………………………….………………..Mobile number (in home country): ………………………………… Home phone: (……...)..…...…........……………………...…Work phone: (….…..)…………………..….……………..……………

GP Name (if available): ……………………………..……………… GP phone(s) ………………………..…………………………

GP address:……………….…………………………………………………………………………….………………………………….

Name of Tour Group: …………………………………………Tour Group leader………………………………..….,.…….…………

Contact Phone No. at Hajj…....…………………………………………………………………………………….…………………….

Date of arrival in Saudi Arabia: …..…./……/2013 Planned date of departure from Saudi Arabia …,...../.……/…………

^day / month / year day / month / year^

**1. Are you currently suffering from any of these symptoms?** (Please mark all that apply)

□Fever; □Cough; □Sore throat; □Runny nose; □Headache; □Muscle pain; □Diarrhoea;

□Breathing difficulty; □Other symptoms (*please specify*)…………………………………………………………………………

**2. Since when have you been suffering from those symptoms?**

Since ……/…..…/2013 (for……….….days)

^day / month / year^

**3. Since you arrived in Saudi Arabia for Hajj 2013, have you visited a hospital / doctor / clinic for cough and fever?**

□No; □Yes, *were you admitted into hospital/clinic?...................................................................................................*

**4. During the last one week, at your hotel room/home how many of your room-mates/household members had coughs, colds, chest infections?**

□None; □Some; □Nearly all

**5. Since arriving in Saudi Arabia have you used facemasks?**

□No; □A few times; □Often

**6. If you wore masks in the last five days, for how many hours each day did you wear masks?**

| *Please provide your answers in this grid* | 5 days ago | 4 days ago | | 3 days ago | 2 days ago | 1 day ago |
| --- | --- | --- | --- | --- | --- | --- |
| About how many hours did you wear masks when you were awake (eg, 4½ hours)? |  |  |  | |  |  |
| Did you use masks during sleep?  *Please write ‘yes’ or ‘no’.* |  |  |  | |  |  |

**…………………………………………………………………………………………………..**

**Doctor Use:** Temp…………..°C Other relevant findings: Nose swab taken: **** No; **** Yes

*Appendix 1 (baseline questionnaire), Version 6: June 26, 2013* Throat swab taken: **** No; **** Yes

**Instructions to Use Facemasks** **English**

1. Clean your hands with soap and water or hand sanitizer before touching the mask.
2. Remove a mask from the box and make sure there are no rips or holes on the inside or outside of the mask.
3. Determine which way around you should wear the mask. The outside has a stiff bendable edge at the top. This bendable edge is designed to be bent over the bridge of the nose.
4. Bring the top of the mask to your nose level and place the top ties over the crown of your head and secure with a bow.
5. Mould or pinch the stiff edge at the top of the mask to the shape of the bridge of your nose.
6. Pull the bottom of the mask over your mouth and chin.
7. Then take the bottom ties, one in each hand, and secure with a bow at the back of your neck.

***Rules for wearing masks***

- Try to avoid touching the front of the mask.
- Change your mask if it is damp, wet or dirty.
- Always clean your hands before and after changing the masks.
- Put used masks in a plastic bag and throw it into a rubbish bin. You will find bins somewhere close to your tent in Mina.

**Hajj Diary English**


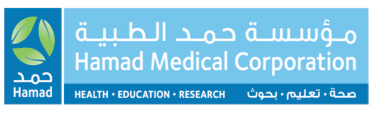

## Hajj Health Diary 13-16 October 8-11 ZUL HIJJA Barcode number

## Name: …………………………………….

Camp number: ……… Tent number: …….
Name of Tour Group: …………………...
Tour Group Leader: …………………….
Tour group leader’s contact phone number: ……………………………………………..

2

1

**Dear Hajj pilgrim,**

Thank you for joining our study.
The Hajj is important to every Muslim. Unfortunately, potentially serious infections such as influenza, coughs, chest infections and rarely Middle East Respiratory Syndrome coronavirus can be acquired and can affect your time at the Hajj. We would like to know if the use of simple facemasks can reduce the frequency of respiratory symptoms among Hajjis.

In this study, there will be two groups of people. One group will be asked to wear facemasks, while the other group will just continue as normal without facemasks. Adult pilgrims (aged 18 years or more) of any gender can participate.

Please tell us about your health during the next 7 days by answering the questions in two health diaries.

This is the first diary, for the days at Mina (13–16 Oct, 2013). The second diary is for 3 days after Mina (17-19 Oct, 2013).

Please take a few minutes before your evening meal each day to record information about your health in the diaries.

If you experience fever and cough or sore throat or runny nose while at Mina, please notify one of our study staff as soon as practically possible. They will take a swab from your nose for later analysis so that we can learn if a virus is causing your symptoms.

On the fourth night at Mina (16 Oct), study staff will collect the first diary. Please continue to answer the questions in your Post-Hajj diary (the 2^nd^ diary) and post it in the self-addressed pre-paid envelope as soon as you reach your home country.

3

4

**Instructions for completing the diary**
Please tick the boxes that are true for you.

For example, if a person was feeling feverish with a runny nose and headache, he or she marks the corresponding boxes:
□ **Feeling feverish**
□ **Cough**
□ **Sore throat**□ **Runny nose**
**□** Headache

If you have any questions about the conduct of the study, please contact:

Dr Harunor Rashid
Phone: +61 2 9845 1489 (Australia)

Email: [harunorr04@gmail.com](mailto:harunorr04@gmail.com)

Dr Haitham El Bashir

Phone: +974 4439 7062 (Qatar)

Email: [HELBASHIR@hmc.org.qa](mailto:HELBASHIR@hmc.org.qa)

Dr Osamah Barasheed
Phone: 0503 362 068 (Saudi mobile)

Email: [barasheedoa@gmail.com](mailto:barasheedoa@gmail.com)

68

5

Day/Month/Year

Day/Month/Year

**Personal Details**
Date of Birth: …./…./……. Age: ….....

Country of Birth: ………………………..

Gender: □Male
 □Female

Home address:............................................………………......................................................................................................................................................................................................

Country.......................................................
Mobile phone number in home country: ……………………………………….……
Other contact number in home country:
……………………………………………

Date arrived in Saudi Arabia: .…./…./2013

**Medical conditions**

Do you have any of the following medical conditions? *Please mark the box that is true with a tick ( )*
□Heart disease: eg. coronary artery disease, congestive heart failure, had heart attack, congenital heart disease.
□Chronic lung diseases: eg. chronic bronchitis, emphysema, chronic obstructive airway disease (COAD), severe asthma, bronchiectasis, cystic fibrosis

□Chronic neurological conditions:
eg. stroke, multiple sclerosis, spinal cord injuries.
□Immune suppression: eg. HIV/AIDS, cancer, leukaemia, long-term (more than 2 weeks) prednisone or methotrexate

7

8

□Diabetes

□Kidney diseases: eg. chronic renal failure, dialysis, nephrotic syndrome

□Other chronic illnesses*(Please name)* …………..…………………..........................................................................................

Are you pregnant? □Yes
 □No

□Not applicable

Do you smoke? □Yes
 □No

Do you have a beard? □Yes
 □No

□Not applicable

Are you on regular medications?

□No

□Yes ……………………………………………

**Influenza vaccination history**

Have you received influenza (flu) vaccine in 2013?

□No

□Yes

If so, where did you get the vaccine?
 □Doctor/GP (including practice nurse)
 □Council clinic

□Mobile clinic (eg. in mosque)
 □Place of work
 □Hospital

□Hajj tour group
 □Travel clinic
 □Other, (*please specify)*. ……………………………………….

10

9

**If you did not get an influenza vaccine, please indicate the reason why:**
 □I didn’t know that the vaccine exists
 □I don’t like injections
 □I am allergic to flu vaccine / allergic to the egg in it
 □I don’t come into contact with people who have flu
 □I rarely get flu
 □I rely on my own natural immunity
 □It costs money
 □I could not get a doctor’s appointment
 □I was too busy
 □The vaccine causes the flu
 □People I know have had bad reactions/ complications
 □The vaccine does not work

Have you received pneumococcal vaccine (for pneumonia) in the last 5 years

□Yes

□No

***If you have fever plus any one of these symptoms please notify study staff in the evening***

12

11

Diary notes made in the evening of Sunday 13/10/2013
**Symptoms:** (*Please mark all of your symptoms)*
□ No symptoms

□ **Feeling feverish**
□ **Cough**
□ **Sore throat**
□ **Runny nose**
□ Headache
□ Muscle pain
□ Shortness of breath
□ Lethargy/ tiredness
□ Vomiting
□ Diarrhoea
□ Sputum/phlegm
□ Shivering
Other symptoms: ……………..……..….. …..……..……..……..……..……..….

Sunday 13/10/2013
During the time that you were awake today, about how many hours did you use a facemask?
 □ None (0 hours)
 □ A few (0-4 hours)
 □ Some (4-8 hours)
 □ Most of the day (8-12 hours)
 □ All of the time

How many facemasks did you use today?
 □ 0
 □ 1-3
 □ 4-6
 □ >6

Did you use facemasks when sleeping last night?
 □ Yes
 □ No

***If you have fever plus any one of these symptoms please notify study staff in the evening***

14

13

Diary notes made in the evening of Monday 14/10/2013
**Symptoms:** *Please mark all of your symptoms*
 □ No symptoms

□ **Feeling feverish**
 □ **Cough**
 □ **Sore throat**
 □ **Runny nose**
 □ Headache
 □ Muscle pain
 □ Shortness of breath
 □ Lethargy/ tiredness
 □ Vomiting
 □ Diarrhoea
 □ Sputum/phlegm
 □ Shivering
Other symptoms: ……………..……..….. …..……..……..……..……..……..……..

Monday 14/10/2013
During the time that you were awake today, about how many hours did you use a facemask?
 □ None (0 hours)
 □ A few (0-4 hours)
 □ Some (4-8 hours)
 □ Most of the day (8-12 hours)
 □ All of the time

How many facemasks did you use today?
 □ 0
 □ 1-3
 □ 4-6
 □ >6

Did you use facemasks when sleeping last night?
 □ Yes
 □ No

***If you have fever plus any one of these symptoms please notify study staff in the evening***

16

15

Diary notes made in the evening of Tuesday 15/10/2013
**Symptoms:** *Please mark all of your symptoms*
□ No symptoms

□ **Feeling feverish**
 □ **Cough**
 □ **Sore throat**
 □ **Runny nose**
 □ Headache
 □ Muscle pain
 □ Shortness of breath
 □ Lethargy/ tiredness
 □ Vomiting
 □ Diarrhoea
 □ Sputum/phlegm
 □ Shivering
Other symptoms: ……………..……..….. …..……..……..……..……..……..……..

Tuesday 15/10/2013
During the time that you were awake today, about how many hours did you use a facemask?
 □ None (0 hours)
 □ A few (0-4 hours)
 □ Some (4-8 hours)
 □ Most of the day (8-12 hours)
 □ All of the time

How many facemasks did you use today?
 □ 0
 □ 1-3
 □ 4-6
 □ >6

Did you use facemasks when sleeping last night?
 □ Yes
 □ No

***If you have fever plus any one of these symptoms please notify study staff in the evening***

18

17

Diary notes made in the evening of Wednesday 16/10/2013
**Symptoms:** *Please mark all of your symptoms*
 □ No symptoms

□ **Feeling feverish**
 □ **Cough**
 □ **Sore throat**
 □ **Runny nose**
 □ Headache
 □ Muscle pain
 □ Shortness of breath
 □ Lethargy/ tiredness
 □ Vomiting
 □ Diarrhoea
 □ Sputum/phlegm
 □ Shivering
Other symptoms: ……………..……..….. …..……..……..……..……..……..……..

Wednesday 16/10/2013

During the time that you were awake today, about how many hours did you use a facemask?
 □ None (0 hours)
 □ A few (0-4 hours)
 □ Some (4-8 hours)
 □ Most of the day (8-12 hours)
 □ All of the time

How many facemasks did you use today?
 □ 0
 □ 1-3
 □ 4-6
 □ >6

Did you use facemasks when sleeping last night?
 □ Yes
 □ No

22

20

21

19

Did you have any problems with using masks? (*please mark all those that are true*)
 □ No problems
 □ Uncomfortable
 □ Difficulty breathing
 □ Inconvenient
 □ Rash
 □ Limitation to social interaction

□ Other: ……..……..……..……..……..

Have you used antiseptic solution/hand rub at any time since arriving in Saudi Arabia?

□Yes

□No

About how many times a day did you wash your hands with soap, antiseptic solution or hand rub (after meal, before meal or touching anything dirty)?

□Each time

□Seldom

□Never

On your way to Saudi Arabia (ie, during your trip to this Hajj) did you visit other countries?

□No □Yes, *list the countries visited* …………………………………………………………………………………………

On your way back to your country of residence (ie, after you leave Saudi Arabia) will you visit any other countries?

□No

□Yes, *please list the countries you will visit on your trip back home*

…………………………………………………………………………………………

Did you visit Medina before Hajj?

□ No

□ Yes, when did you return to Mecca? Date…..…./…..…/…………

If you have NOT already visited Medina do you plan to do so at the end of the Hajj?

□No □ Yes

Thank you for completing this diary.

Rest assured that all information you provide is confidential.

Please return this diary to study staff at on the evening of the 4^th^ day.

If you forget to return your first diary to study staff before you left Mina, please post it along with your Post-Hajj diary in the self-addressed envelope provided.

**Post-Hajj Diary English**


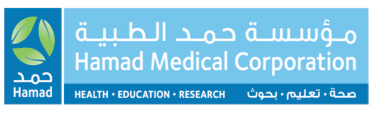

## Post-Hajj Health Diary 17-19 October 12-14 ZUL HIJJA Barcode number

## Name: ………..………………………….

Camp number: ……… Tent number: …....
Name of Tour Group:…..………………...
Tour Group Leader:………...…………….
Tour Group Leader’s Saudi phone number:

……………………....................................

**Dear Pilgrim**,

Thank you for continuing with our study to find out how well facemasks reduce the frequency of respiratory symptoms among Hajjis. This work forms part of our long term goal to reduce levels of illnesses at Hajj and improve healthcare services provided to pilgrims.
In this Post-Hajj diary, please tell us about your health for 3 days after Hajj (17-19 Oct).
A self-addressed envelope is supplied in the study pack for convenient postal delivery of the diary to our research group.
We hope you have enjoyed your time at Hajj 2013. 1

**Personal Details**
Date of Birth: …./…./……. Age: ….....

Country of Birth: ………………………..

Gender: □ Male
 □ Female

Home address: …………………………...
…………………………………………...............................................................................................................................................Country:.......................................................
Mobile phone number in home country: ……………………………………………
Other contact number: …………………..
Date of departure from Saudi Arabia:…...../…....../2013

2

3

4

**Instructions for completing the diary**
Please tick the boxes that are true for you.

For example, if a person was feeling feverish with a runny nose and headache, he or she marks the corresponding boxes:
□ **Feeling feverish**
□ **Cough**
□ **Sore throat**□ **Runny nose**
**□** Headache

If you have any questions about the conduct of the study, please contact:

Dr Harunor Rashid
Phone: +61 29845 1489 (Australia)

Email: [harunorr04@gmail.com](mailto:harunorr04@gmail.com)

Dr Haitham El Bashir

Phone: +974 4439 7062 (Qatar)

Email: [HELBASHIR@hmc.org.qa](mailto:HELBASHIR@hmc.org.qa)

Dr Osamah Barasheed
Phone: 0503 362 068 (Saudi mobile)

Email: [barasheedoa@gmail.com](mailto:barasheedoa@gmail.com)

Diary notes made in the evening of Thursday 17/10/2013
**Symptoms:** *Please mark all of your symptoms*
 □ No symptoms
 □ **Feeling feverish**
 □ **Cough**
 □ **Sore throat**
 □ **Runny nose**
 □ Headache
 □ Muscle pain
 □ Shortness of breath
 □ Lethargy/tiredness
 □ Vomiting
 □ Diarrhoea
 □ Sputum/phlegm
 □ Shivering
Other symptoms: ……………..……...................................….

5

Thursday 17/10/2013
During the time that you were awake today, about how many hours did you use a facemask?
 □ None (0 hours)
 □ A few (0-4 hours)
 □ Some (4-8 hours)
 □ Most of the day (8-12 hours)
 □ All of the time

Did you use facemasks when sleeping last night?
 □ Yes
 □ No

How many facemasks did you use today?
 □ 0
 □ 1-3
 □ 4-6
 □ >6

6

Diary notes made in the evening of Friday 18/10/2013
**Symptoms:** *Please mark all of your symptoms*

□ No symptoms
 □ **Feeling feverish**
 □ **Cough**
 □ **Sore throat**
 □ **Runny nose**
 □ Headache
 □ Muscle pain
 □ Shortness of breath
 □ Lethargy/tiredness
 □ Vomiting
 □ Diarrhoea
 □ Sputum/phlegm
 □ Shivering
Other symptoms: ……………..……..….....
 7 7

Friday 18/10/2013
During the time that you were awake today, about how many hours did you use a facemask?
 □ None (0 hours)
 □ A few (0-4 hours)
 □ Some (4-8 hours)
 □ Most of the day (8-12 hours)
 □ All of the time

Did you use facemasks when sleeping last night?
 □ Yes
 □ No

How many facemasks did you use today?
 □ 0
 □ 1-3
 □ 4-6
 □ >6
 8
 8

Diary notes made in the evening of Saturday 19/10/2013
**Symptoms:** *Please mark all of your symptoms*
 □ No symptoms

□ **Feeling feverish**
 □ **Cough**
 □ **Sore throat**
 □ **Runny nose**
 □ Headache
 □ Muscle pain
 □ Shortness of breath
 □ Lethargy/tiredness
 □ Vomiting
 □ Diarrhoea
 □ Sputum/phlegm
 □ Shivering
Other symptoms: ……………..……..…..

9

Saturday 19/10/2013
During the time that you were awake today, about how many hours did you use a facemask?
 □ None (0 hours)
 □ A few (0-4 hours)
 □ Some (4-8 hours)
 □ Most of the day (8-12 hours)
 □ All of the time

Did you use facemasks when sleeping last night?
 □ Yes
 □ No

How many facemasks did you use today?
 □ 0
 □ 1-3
 □ 4-6
 □ >6
 10

Did you have any problems with using masks? (*Please mark all those that are true*)
 □ No problems
 □ Uncomfortable
 □ Difficulty breathing
 □ Inconvenient
 □ Rash
 □ Limitation to social interaction/
 communication
 □ Other: ……..……..……..……..……..
 ……..……..……..……..……..……..…..

11

Thank you very much for participating in this study.
Rest assured that all information you provide is confidential.

Please place this diary in the self-addressed envelope and post it at the earliest convenience after your home arrival.

12

**Baseline Questionnaire Arabic**

**دراسة عشوائية بالقرعة لاختبار فعالية استخدام الكمامات للوقاية من الأمراض التنفسية المعدية بين الحجاج أثناء موسم الحج**

**_____________________________________________________________________________**

الاسم.................................اللقب..............................تاريخ الميلاد ............/............./..............(العمر..........)

بلد الإقامة ............................................بلد الميلاد......................................الجنس :ذكر*ρ* / أنثى****

العِرق........................................................المهنة ..............................................................................

العنوان في بلد الإقامة............................................................................................................................

....................................................................................................................................................

البريد الالكتروني : ................................................رقم الهاتف الجوّال في بلد الإقامة.........................................

رقم هاتف المنزل ........................................................رقم هاتف العمل ....................................................

اسم طبيبك (إن وجد) ...................................................هاتف طبيبك .........................................................

عنوان طبيبك ....................................................................................................................................

اسم وكالة السفر........................................................... اسم مندوب وكالة السفر............................................

رقم هاتفك أثناء الحجّ.............................................................................................................................

تاريخ الوصول للمملكة العربية السعودية......./........../..... التاريخ المُتوقّع لمغادرة المملكة العربية السعودية......../......./......

1. هل تمّ تطعيمك بأيّ من التطعيمات التالية ؟ (بالإمكان اختيار أكثر من إجابة):

   ****تطعيم الانفلونزا لسنة (2013)****تطعيم المكوّرات العنقودية (الالتهاب الرئوي) في أيّ من الخمس سنوات السابقة****أيّ تطعيم اخر...............
2. هل تعاني أيّ من الأمراض التالية ؟ (بالإمكان اختيار أكثر من إجابة):

   ****مرض قلبي مثل (النوبات القلبيّة أو الجلطات , أمراض شرايين , فشل عضلات القلب , تشوّهات خلقيّة)
   ****مرض صدري (رئويّ) مثل (التهاب القصبات الهوائيّة المزمن , نفاخ رئوي , ربو حاد , توسّع القصبات , التليّف الكيسي )
   **** داء السُّكري
   ****مرض كلوي مثل الفشل الكلوي
   ****مرض في الجهاز العصبي مثل الجلطات الدماغية , تصلّب لويحي , إصابة في النخاع الشوكي ****أمراض تؤدي لنقص المناعة مثل ( أورام , سرطان الدم , استعمال أدوية - لمدة أكثر من اسبوعين- تؤدي لنقص المناعة مثل prednisone أو methotrexate
   **** أي مرض آخر (يرجى التحديد)................................................................................................
3. هل تتناول دواء معيّن بانتظام ؟ **** لا **** نعم (يرجى تحديد اسم الدواء)....................................
4. هل تدخّن (أو هل مارست التدخين في الفترة ماقبل السفر للحج ) ؟ **** لا **** نعم
5. هل تعاني حاليّاً من أيّ من الأعراض التاليّة ؟ (بإمكانك اختيار أكثر من أجابة):

   **** ارتفاع في درجة الحرارة****كُحّة**** التهاب في الحلق ****سَيَلان في الأنف****صداع ****ألم في العضلات****اسهال****ضعف
   أي عَرَض آخر (يُرجى التحديد) ......................................
6. إذا كنت تعاني من أي من الأعراض السابقة يُرجى تحديد الزمن الذي بدأ فيه العَرَض
   منذ تاريخ ......../........./......... (لمدّة...............يوم)
7. منذ وصولك للملكة العربية السعودية لموسم الحج 2013 , هل سبق وأن زرت أي مستشفى أو طبيب أو عيادة بسبب أعراض تشبه الزكام ؟
   **** لا **** نعم (هل سبق لك الإيواء في مستشفى أو عيادة ؟) ...................................
8. كم عدد المصاحبين لك في غرفة الفندق ممن عانى من (كحّة أو زكام أو برد أو التهاب صدري) ؟
   ****لا أحد**** بعضهم****تقريباً كلّهم
9. هل استعملت محلول مطهّر / معقّم لليدين خلال موسم الحج 2013 ؟
   **** لا **** نعم (كم مرة في اليوم؟) ................................................................
10. كم مرّة في النهار تغسل يديك بالصابون خلال موسم الحج 2013 ؟ (يرجى عدم احتساب الوضوء)
    **** 0 ****1-2****3-4****<5 مرات في اليوم
11. هل استعملت (كمامة الوجه) خلال موسم الحج 2013 ؟
    **** لا **** نعم

    إذا استعملت (كمامة الوجه) خلال الخمسة أيام السابقة , فكم عدد الساعات التي لبست فيها الكمامة في اليوم الواحد ؟

| يُرجى الإجابة في هذا الجدول | الـ5 أيام السابقة | الـ4 أيام السابقة | الـ3 أيام السابقة | اليومان السابقان | اليوم السابق |
| --- | --- | --- | --- | --- | --- |
| تقريباً كم عدد الساعات التي ارتديت فيها كمامة الوجه وأنت يقِظ (مثال , 4½ساعة؟) |  |  |  |  |  |
| هل استعملت الكمامة أثناء النّوم ؟ يرجى الإجابة بـ"نعم" أو "لا" |  |  |  |  |  |

1. في طريقك للسعودية (رحلة الحج) هل قمت بزيارة بلدان أُخرى ؟
   **** لا **** نعم (يُرجى تحديد البُلدان) ...........................................................................
2. أثناء رحلتك للرجوع لبلد الإقامة (بعد مغادرة المملكة العربية السعودية) هل تنتوي زيارة بُلدان أُخرى ؟
   **** لا **** نعم (يُرجى تحديد البُلدان) ............................................................................
3. هل زُرت (المدينة) قبل الحج ؟ ****لا **** نعم , متى رجعت لمكّة؟ التاريخ .........../............/.........
4. إذا لم تكُن قد زرت (المدينة) فهل تخطط لزيارتها بعد الحج ؟ ****لا **** نعم

شكراً لإتمامك هذا الاستقصاء

______________________________________________________________________________

ملاحظات الطبيب : درجة الحرارة ..............°C أعراض أُخرى مهمّة................................

**Instructions to use facemask Arabic**

**تعليمات استخدام الكمامة:**

1. **اغسل يديك جيداً بالماء والصابون أو بمطهر اليدين قبل لمس الكمامة.**
2. **اخرج كمامة من العلبة وتأكد من عدم وجود اي تمزق او ثقوب في الوجه الداخلي أوالخارجي للكمامة.**
3. **حدد الطريقة الصحيحة للبس الكمامة. يحتوي الوجه الخارجي للكمامة على حد علوي قاسي قابل للثني. صُمم الحد القابل للثني لينحني على قصبة الانف.**
4. **اجعل الحد الاعلى من الكمامة على مستوى الأنف وضع الاربطة العلوية على الرأس واحكمها بانحناء.**
5. **ضيق او اضغط الحد القاسي في اعلى الكمامة على قصبة الانف.**
6. **اسحب الحد السفلي من الكمامة على الفم والذقن.**
7. **ثم خذ الاربطة السفلية واحكمها بانحناء خلف الرقبة.**

قواعد لبس الكمامة:

- **حاول تجنب لمس الوجه الخارجي للكمامة.**
- **غير كمامتك اذا ترطبت او ابتلت او اتسخت.**
- **نظف يديك دائما قبل وبعد تغيير الكمامات.**
- **ضع الكمامات المستخدمة في كيس بلاستيك ثم القه في صندوق النفايات. سوف تجد صندوق نفايات في مكان ما قريباً من خيمتك في منى.**

**Hajj Diary Arabic**


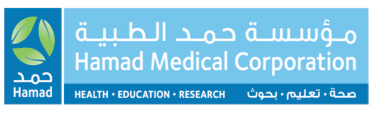

## يومية الحج الصحية 13-16 أكتوبر 8-11 ذو الحجة رقم الباركود

## الاسم: …………………………………….

رقم المخيم: ……… رقم الخيمة: …….
اسم مجموعة الحج: …………………...
اسم مسئول الحملة: …………………….
رقم مسئول الحملة: ……………………………………………..

2

1

**عزيزي الحاج,**

شكرا لمشاركتك معنا في هذا البحث

يعتبر الحج حدث مهم لكل مسلم . لكن للأسف قد يتعطل هذا الحدث المهم بالاصابة ببعض الالتهابات مثل الانفلونزا، والتهابات الصدر، والسعال، ونزلات البرد ونادرا جدا السارس . ونود أن نرى إمكانية منع هذه العدوى بإجراءات بسيطة مثل لبس الكمامات (أقنعة الوجه).

في هذه الدراسة، سيكون هناك مجموعتين من الناس. وسيطلب من مجموعة واحدة ارتداء الكمامات ، في حين أن مجموعة أخرى سوف تستمر فقط كالمعتاد دون كمامات . يمكن للحجاج البالغين (الذين تتراوح أعمارهم بين 18 سنة أو أكثر) من أي من الجنسين المشاركة.

من فضلك أخبرنا عن صحتك خلال السبعة ايام القادمة عن طريق الإجابة على الأسئلة في اثنين من اليوميات الصحية.

هذه هي أول يومية ، لأيام منى في الفترة (13-16 أكتوبر، 2013). أما اليومية الثانية فهي لما بعد منى في الفترة (17-19 أكتوبر 2013).

يرجى أخذ بضع دقائق قبل وجبة المساء الخاصة بك كل يوم لتسجيل المعلومات عن صحتك في اليوميات.

إذا واجهت الحمى والسعال أو التهاب الحلق أو سيلان الأنف وأنت في منى، الرجاء إبلاغ أحد أعضاء فريق البحث في أقرب وقت ممكن . وعليها فسيقوم بأخذ مسحة من الأنف أو الحلق لتحليلها لاحقا حتى نتمكن من معرفة ما هو الفيروس المسبب للأعراض التي أصابتك.

وفي الليلة الرابعة في منى (16 أكتوبر)، سيقوم فريق الدراسة بجمع هذه اليوميات الأولى. يرجى الاستمرار في الإجابة على الأسئلة الخاصة بك في اليوميات الثانية (مذكرات 2) وبعد ذلك نرجو إرسالها عبر البريد عن طريق المظاريف المسبقة الدفع ، والمعنونة مسبقا حالما تصل بلدك.

3

4

**تعليمات لتعبئة اليوميات الصحية**
يرجى وضع إشارة صح على الإجابات الصحيحة.

على سبيل المثال ، إذا كان الشخص يشعر بالحمى مع سيلان الأنف والصداع ، فإنه يضع إشارة على المربعات المربعات المقابلة للخيارات الصحيحة:
□ **تشعر بالحمى**
□ **سعال**
□ **التهاب الحلق**□ **سيلان الأنف**
**□** صداع

إذا كان لديك أية استفسارات أو تساؤلات حول المشاركة في البحث فلا تتردد بالاتصال بنا

الدكتور هارون الرشيد
تلفون : +61 2 9845 1489 (Australia)

Email: [harunorr04@gmail.com](mailto:harunorr04@gmail.com)

الدكتور هيثم البشير

تلفون : +974 4439 7062 (Qatar)

Email: [HELBASHIR@hmc.org.qa](mailto:HELBASHIR@hmc.org.qa)

الدكتور أسامة بارشيد
تلفون : 0503 362 068 (Saudi mobile)

Email: [barasheedoa@gmail.com](mailto:barasheedoa@gmail.com)

يوم/ شهر/سنة

68

5

يوم/ شهر /سنة

**معلومات شخصية**
تاريخ الميلاد : …./…./……. العمر: ….....

بلد الميلاد : ………………………..

الجنس : □ذكر
 □أنثى

عنوان المنزل:............................................………………......................................................................................................................................................................................................

الدولة.......................................................
رقم الهاتف الجوال في بلدك : ……………………………………….……
رقم اتصال آخر في بلدك :
……………………………………………

تاريخ الوصول للسعودية : .…./…./2013

**الحالة الطبية العامة**

هل تعاني من أي من هذه الأمراض؟

*يرجى وضع علامة صح على الخيار الصحيح ( )*
□**مرض في القلب** : على سبيل المثال: مرض الشريان التاجي، وفشل القلب الاحتقاني، والنوبات القلبية، وأمراض القلب الخلقية.
□مرض رئوي مزمن : على سبيل المثال: التهاب الشعب الهوائية المزمن، وانتفاخ الرئة، وأمراض الشعب الهوائية الانسدادي المزمن (COAD)، الربو الحاد، توسع القصبات، والتليف الكيسي

□مرض مزمن في المخ والأعصاب :
على سبيل المثال: السكتة الدماغية، والتصلب المتعدد، وإصابات الحبل الشوكي.
□ضعف في المناعة : على سبيل المثال: فيروس نقص المناعة البشرية / الإيدز، والسرطان، وسرطان الدم، تأخذ علاج بريدنيزون أو الميثوتريكسيت على مدى طويل (أكثر من أسبوعين)

7

8

□مرض السكري

□أمراض الكلى : على سبيل المثال: الفشل الكلوي المزمن، وغسيل الكلى، والمتلازمة الكلوية

□أمراض مزمنة أخرى *(نرجو كتابتها )* …………..…………………..........................................................................................

هل أنتِ حامل؟ □نعم
 □لا

□ غير قابل للتطبيق

هل تدخن ؟ □نعم

□لا

هل لديك لحية؟ □نعم
 □لا

□ غير قابل للتطبيق

هل أنت على علاج بصفة منتظمة؟

□لا

□نعم ……………………………………………

**تاريخ التطعيم ضد الانفلونزا**

هل أخذت تطعيم (لقاح) ضد الانفلونزا لعام 2013م ؟

□لا

□نعم

إذا كنت أخذته ، فأين حصلت على التطعيم؟
 □ (بما في ذلك الممرضة الممارسة) طبيب العائلة
 □عيادة المجلس البلدي

□عيادة متنقلة (مثلا في المسجد)
 □مكان العمل
 □المستشفى

□مجموعة (حملة) الحج
 □عيادة السفر
 □أخرى , (*نرجو التحديد)*. ……………………………………….

10

9

**إذا لم تحصل على تطعيم (لقاح) الانفلونزا ، نرجو ذكر السبب لذلك:**
 □لم أعلم بوجود التطعيم ضد الانفلونزا
 □لا أحب وخز الإبر
 □لدي حساسية من تطعيم الانفلونزا أو من البيض الذي يحتويه
 □ليس لدي احتكاك أو اتصال مباشر مع المصابين بالانفلونزا
 □نادرا ما أصاب بالانفلونزا
 □أعتمد على مناعتي الطبيعية
 □لأنه يكلفني المال
 □لم أستطع الحصول على موعد مع الطبيب
 □كنت مشغولا جدا
 □أعتقد أن التطعيم يسبب الانفلونزا
 □الناس الذين أعرفهم لديهم ردود فعل (مضاعفات) سيئة بسبب التطعيم
 □أعتقد أن التطعيم ليس فعالا

**هل أخذت لقاح المكورات الرئوية (للالتهاب الرئوي) خلال الخمس سنوات الأخيرة؟**

□نعم

□لا

**إذا كان لديك حمى بالإضافة إلى أي واحد من هذه الأعراض، الرجاء إبلاغ فريق البحث**

12

11

الملاحظات اليومية لمساء يوم الأحد 13/10/2013
**الأعراض:** (*نرجو تحديد كل الأعراض التي لديك)*
□ لا توجد أعراض

□ **تشعر بحمى**
□ **سعال**
□ **التهاب الحلق**
□ **سيلان الأنف**
□ صداع
□ ألم في العضلات
□ ضيق في التنفس
□ خمول أو إرهاق
□ قيء أو استفراغ
□ إسهال
□ بلغم
□ رعشة
أعراض أخرى: ……………..……..….. …..……..……..……..……..……..….

الأحد 13/10/2013
كم ساعة تقريبا استخدمت الكمامات خلال هذا اليوم ؟

□ ولامرة (0 ساعة)
 □ قليل (0-4 ساعات)
 □ أحيانا (4-8 ساعات)
 □ أغلب اليوم (8-12 ساعات)
 □ طوال اليوم

كم عدد الكمامات التي استخدمتها خلال هذا اليوم؟
 □ 0
 □ 1-3
 □ 4-6
 □ >6

هل استخدمت الكمامة وأنت نائم ليلة البارحة؟
 □ نعم
 □ لا

**إذا كان لديك حمى بالإضافة إلى أي واحد من هذه الأعراض، الرجاء إبلاغ فريق البحث**

14

13

الملاحظات اليومية لمساء يوم الاثنين 14/10/2013
**الأعراض:** (*نرجو تحديد كل الأعراض التي لديك)*
□ لا توجد أعراض

□ **تشعر بحمى**
□ **سعال**
□ **التهاب الحلق**
□ **سيلان الأنف**
□ صداع
□ ألم في العضلات
□ ضيق في التنفس
□ خمول أو إرهاق
□ قيء أو استفراغ
□ إسهال
□ بلغم
□ رعشة
أعراض أخرى: ……………..……..….. …..……..……..……..……..……..……..

الاثنين 14/10/2013
كم ساعة تقريبا استخدمت الكمامات خلال هذا اليوم ؟

□ ولامرة (0 ساعة)
 □ قليل (0-4 ساعات)
 □ أحيانا (4-8 ساعات)
 □ أغلب اليوم (8-12 ساعات)
 □ طوال اليوم

كم عدد الكمامات التي استخدمتها خلال هذا اليوم؟
 □ 0
 □ 1-3
 □ 4-6
 □ >6

هل استخدمت الكمامة وأنت نائم ليلة البارحة؟
 □ نعم
 □ لا

**إذا كان لديك حمى بالإضافة إلى أي واحد من هذه الأعراض، الرجاء إبلاغ فريق البحث**

16

15

الملاحظات اليومية لمساء يوم الثلاثاء 15/10/2013
**الأعراض:** (*نرجو تحديد كل الأعراض التي لديك)*
□ لا توجد أعراض

□ **تشعر بحمى**
□ **سعال**
□ **التهاب الحلق**
□ **سيلان الأنف**
□ صداع
□ ألم في العضلات
□ ضيق في التنفس
□ خمول أو إرهاق
□ قيء أو استفراغ
□ إسهال
□ بلغم
□ رعشة
أعراض أخرى: ……………..……..….. …..……..……..……..……..……..……..

الثلاثاء 15/10/2013
كم ساعة تقريبا استخدمت الكمامات خلال هذا اليوم ؟

□ ولامرة (0 ساعة)
 □ قليل (0-4 ساعات)
 □ أحيانا (4-8 ساعات)
 □ أغلب اليوم (8-12 ساعات)
 □ طوال اليوم

كم عدد الكمامات التي استخدمتها خلال هذا اليوم؟
 □ 0
 □ 1-3
 □ 4-6
 □ >6

هل استخدمت الكمامة وأنت نائم ليلة البارحة؟
 □ نعم
 □ لا

**إذا كان لديك حمى بالإضافة إلى أي واحد من هذه الأعراض، الرجاء إبلاغ فريق البحث**

18

17

الملاحظات اليومية لمساء يوم الأربعاء 16/10/2013
**الأعراض:** (*نرجو تحديد كل الأعراض التي لديك)*
□ لا توجد أعراض

□ **تشعر بحمى**
□ **سعال**
□ **التهاب الحلق**
□ **سيلان الأنف**
□ صداع
□ ألم في العضلات
□ ضيق في التنفس
□ خمول أو إرهاق
□ قيء أو استفراغ
□ إسهال
□ بلغم
□ رعشة
أعراض أخرى: ……………..……..….. …..……..……..……..……..……..……..

الأربعاء 16/10/2013
كم ساعة تقريبا استخدمت الكمامات خلال هذا اليوم ؟

□ ولامرة (0 ساعة)
 □ قليل (0-4 ساعات)
 □ أحيانا (4-8 ساعات)
 □ أغلب اليوم (8-12 ساعات)
 □ طوال اليوم

كم عدد الكمامات التي استخدمتها خلال هذا اليوم؟
 □ 0
 □ 1-3
 □ 4-6
 □ >6

هل استخدمت الكمامة وأنت نائم ليلة البارحة؟
 □ نعم
 □ لا

22

20

21

19

هل عانيت من أية مشاكل عند استخدامك للكمامات ؟ (*نرجو تحديد كل ما تراه صحيحا*)
 □ لاتوجد مشاكل
 □ غير مريحة
 □ صعوبة في التنفس
 □ طفح جلدي
 □ تحد من التواصل الاجتماعي

□ أخرى: ……..……..……..……..……..

**هل استخدمت محلول مطهر / معقم اليد في أي وقت منذ وصولك إلى المملكة العربية السعودية ؟**

□نعم

□لا

**كم مرة تقريبا غسلت فيها يديك بالصابون أو بمطهر/معقم اليدين خلال هذا اليوم؟** (بعد الطعام, أو قبل الطعام أو بعد لمس أي شيء متسخ مثلا)?

□**كل مرة**

**□نادرا**

□ولا مرة

هل زرت أي دولة أخرى في طريقك لرحلة الحج إلى المملكة العربية السعودية؟

□لا □نعم , (نرجو كتابة اسم الدولة التي زرتها) …………………………………………………………………………………………

هل ستزور دولة أخرى في طريق عودتك إلى بلادك بعد الحج ؟

□لا

□نعم , (نرجو كتابة اسم الدولة التي ستزورها)

…………………………………………………………………………………………

هل زرت المدينة المنورة قبل الحج؟

□ لا

□ نعم , *(متى عدت منها إلى مكة؟)* التاريخ…..…./…..…/…………

إذا لم تكن قد ذهبت للمدينة المنورة قبل الحج ، فهل لديك نية لزيارتها بعد الحج؟

□لا □ نعم

شكرا لتعاونك معنا في تعبئة هذه اليومية .

اطمئن بأن كافة المعلومات التي قدمتها ستكون سرية .

نرجو إعادة هذه اليومية لأحد أعضاء فريق البحث في مساء اليوم الرابع .

إذا نسيت إعادة هذه اليومية لفريق البحث ، فيمكنك إرسالها بالبريد مع اليومية الثانية (يومية مابعد الحج) عبر المظروف الذي لديك .

**Post-Hajj Diary Arabic**


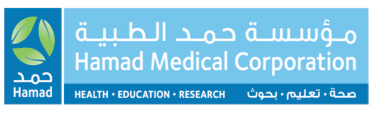

## يومية مابعد الحج الصحية 17-19 أكتوبر 12-14 ذو الحجة رقم الباركود

## الاسم: …………………………………….

رقم المخيم: ……… رقم الخيمة: …….
اسم مجموعة الحج: …………………...
اسم مسئول الحملة: …………………….
رقم مسئول الحملة: ……………………………………………..

**عزيزي الحاج**

نشكرك على مواصلة مشاركتك في دراستنا لمعرفة مدى فاعلية الكمامات في الحد من انتشار التهابات الصدر بين الحجاج.

يشكل هذا العمل جزءا من هدفنا على المدى الطويل للحد من مستويات الأمراض في الحج وتحسين خدمات الرعاية الصحية المقدمة لحجاج بيت الله الحرام.

في هذه المذكرة (يومية مابعد الحج)، من فضلك أخبرنا عن صحتك لمدة 3 أيام بعد الحج 17-19 أكتوبر .

وسيتم تزويدك بظرف مسبق الدفع وعليه عنوان مركز الأبحاث لتسهيل إرساله لنا بالبريد بعد تعبئته.

نأمل لكم وقتا ممتعا ومفيدا في حج عام 2013.

1

**معلومات شخصية**
تاريخ الميلاد : …./…./……. العمر: ….....

بلد الميلاد : ………………………..

الجنس : □ذكر
 □أنثى

عنوان المنزل:............................................………………..............................................................................................................................

الدولة.......................................................
رقم الهاتف الجوال في بلدك : ……………………………………….……
رقم اتصال آخر في بلدك :
……………………………………………
تاريخ مغادرة السعودية : .…./…./2013

2

3

4

**تعليمات لتعبئة اليوميات الصحية**
يرجى وضع إشارة صح على الإجابات الصحيحة.

على سبيل المثال ، إذا كان الشخص يشعر بالحمى مع سيلان الأنف والصداع ، فإنه يضع إشارة على المربعات المربعات المقابلة للخيارات الصحيحة:
□ **تشعر بالحمى**
□ **سعال**
□ **التهاب الحلق**□ **سيلان الأنف**
**□** صداع

إذا كان لديك أية استفسارات أو تساؤلات حول المشاركة في البحث فلا تتردد بالاتصال بنا

الدكتور هارون الرشيد
تلفون : +61 2 9845 1489 (Australia)

Email: [harunorr04@gmail.com](mailto:harunorr04@gmail.com)

الدكتور هيثم البشير

تلفون : +974 4439 7062 (Qatar)

Email: [HELBASHIR@hmc.org.qa](mailto:HELBASHIR@hmc.org.qa)

الدكتور أسامة بارشيد
تلفون : 0503 362 068 (Saudi mobile)

Email: [barasheedoa@gmail.com](mailto:barasheedoa@gmail.com)

الملاحظات اليومية لمساء يوم الخميس 17/10/2013
**الأعراض:** (*نرجو تحديد كل الأعراض التي لديك)*
□ لا توجد أعراض

□ **تشعر بحمى**
□ **سعال**
□ **التهاب الحلق**
□ **سيلان الأنف**
□ صداع
□ ألم في العضلات
□ ضيق في التنفس
□ خمول أو إرهاق
□ قيء أو استفراغ
□ إسهال
□ بلغم
□ رعشة
أعراض أخرى: ……………..……..…..

5

الخميس 17/10/2013
كم ساعة تقريبا استخدمت الكمامات خلال هذا اليوم ؟

□ ولامرة (0 ساعة)
 □ قليل (0-4 ساعات)
 □ أحيانا (4-8 ساعات)
 □ أغلب اليوم (8-12 ساعات)
 □ طوال اليوم

كم عدد الكمامات التي استخدمتها خلال هذا اليوم؟
 □ 0
 □ 1-3
 □ 4-6
 □ >6

هل استخدمت الكمامة وأنت نائم ليلة البارحة؟
 □ نعم
 □ لا
 6

الملاحظات اليومية لمساء يوم الجمعة 18/10/2013
**الأعراض:** (*نرجو تحديد كل الأعراض التي لديك)*
□ لا توجد أعراض

□ **تشعر بحمى**
□ **سعال**
□ **التهاب الحلق**
□ **سيلان الأنف**
□ صداع
□ ألم في العضلات
□ ضيق في التنفس
□ خمول أو إرهاق
□ قيء أو استفراغ
□ إسهال
□ بلغم
□ رعشة
أعراض أخرى: ……………..……..…..
 7 7

الجمعة 18/10/2013
كم ساعة تقريبا استخدمت الكمامات خلال هذا اليوم ؟

□ ولامرة (0 ساعة)
 □ قليل (0-4 ساعات)
 □ أحيانا (4-8 ساعات)
 □ أغلب اليوم (8-12 ساعات)
 □ طوال اليوم

كم عدد الكمامات التي استخدمتها خلال هذا اليوم؟
 □ 0
 □ 1-3
 □ 4-6
 □ >6

هل استخدمت الكمامة وأنت نائم ليلة البارحة؟
 □ نعم
 □ لا

8

الملاحظات اليومية لمساء يوم السبت 19/10/2013
**الأعراض:** (*نرجو تحديد كل الأعراض التي لديك)*
□ لا توجد أعراض

□ **تشعر بحمى**
□ **سعال**
□ **التهاب الحلق**
□ **سيلان الأنف**
□ صداع
□ ألم في العضلات
□ ضيق في التنفس
□ خمول أو إرهاق
□ قيء أو استفراغ
□ إسهال
□ بلغم
□ رعشة
أعراض أخرى: ……………..……..….. 9

السبت 19/10/2013
كم ساعة تقريبا استخدمت الكمامات خلال هذا اليوم ؟

□ ولامرة (0 ساعة)
 □ قليل (0-4 ساعات)
 □ أحيانا (4-8 ساعات)
 □ أغلب اليوم (8-12 ساعات)
 □ طوال اليوم

كم عدد الكمامات التي استخدمتها خلال هذا اليوم؟
 □ 0
 □ 1-3
 □ 4-6
 □ >6

هل استخدمت الكمامة وأنت نائم ليلة البارحة؟
 □ نعم
 □ لا

10

هل عانيت من أية مشاكل عند استخدامك للكمامات ؟ (*نرجو تحديد كل ما تراه صحيحا*)
 □ لاتوجد مشاكل
 □ غير مريحة
 □ صعوبة في التنفس
 □ طفح جلدي
 □ تحد من التواصل الاجتماعي

□ أخرى: ……..…......................................................…..……..……..……..

11

شكرا لتعاونك معنا في تعبئة هذه اليومية .

اطمئن بأن كافة المعلومات التي قدمتها ستكون سرية .

نرجو إرسال هذه اليومية عبر البريد بواسطة المظروف مسبق الدفع والذي تم كتابة عنوان مركز الأبحاث عليه في أسرع وقت حين وصولك لبلدك بعد رحلة الحج .

12

**Consent Form English**

***Research Title:*** *Cluster-randomized controlled trial to test the effectiveness of facemasks in preventing respiratory virus infection among Hajj pilgrims*

***Principal Investigators:*** Dr Haitham El Bashir, Dr Harunor Rashid

We invite you to take part in research at Hajj pilgrimage. We wish to find out if giving masks to Hajj pilgrims stops transmission of influenza and other respiratory viruses. You are being asked to join in this study because you reside in Australia, Saudi Arabia or Qatar, and over 18 years old. We aim at recruiting 7000 pilgrims.

If you agree to join this study, we will ask you for some information about you and your health, and give you a pair of diaries (one to be completed at Hajj, and the other back home). In some tents but not in others we shall provide masks free of cost. We will then follow what happens to you.

If you have a cold, a cough, a chest infection or flu please seek out one of our team who will visit your tent at least once a day. If you are ill we will take swabs from your nose (or throat if that’s more convenient to you) to test for viral infection.

We expect the only risks to be a tickling sensation, sneeze and may have watering from eyes if a swab is collected from your nose or throat.

If you have flu symptoms, we may give you standard painkillers and other medications to relieve your pain and fever.

You do not have to join this study. It is up to you. You can say okay now and change your mind later. If you say no or change your mind, there will be no effect on you.

Before you say **YES or NO** to being in this study, we will answer any questions you have. If you join the study, you can ask questions at any time. Just tell the researcher that you have a question.

If you have any questions about this study please feel free to contact

- Dr Osamah Barasheed, Mobile: +966 503 362 068 (Saudi Arabia), Email: [barasheedoa@gmail.com](mailto:barasheedoa@gmail.com)
- Dr Haitham El Bashir, Tel: +974 4439 7062 (Qatar), Email: [helbashir@hmc.org.qa](mailto:helbashir@hmc.org.qa)
- Dr Harunor Rashid, Tel: +61 29845 1489 (Australia) , Email: [harunor.rashid@health.nsw.gov.au](mailto:harunor.rashid@health.nsw.gov.au)

|  | | |
| --- | --- | --- |
| Signature of volunteer | Person Obtaining Consent | |
| I voluntarily agree to join the research described in this form.  ---------------------------------(Printed Name of Volunteer)  ---------------------------------(Signature of Volunteer)  ---------------------------------(Date) | *I document that:*   - *I (or another member of the research team) have explained this research to the volunteer.* - *I have personally evaluated the volunteer’s understanding of the research and obtained their voluntary agreement.*   *----------------------------------- (*Printed Name of Person Obtaining Consent)  ---------------------------- ------(Signature of Person Obtaining Consent)  -----------------------------------(Date) | |

**Consent Form English**

**Consent Form Arabic**

**نموذج موافقة المشاركة بالبحث**

**عنوان البحث: دراسة عشوائية بالقرعة لاختبار فعالية استخدام الكمامات (قناع الوجه) للوقاية من الأمراض التنفسية المعدية بين الحجاج أثناء موسم الحج**

**الباحث الرئيسي: د. هيثم البشير ، د. هارون الرشيد .**

نود أن نطلعك على دراسة بحثية نقوم بها . لمعرفة المزيد عن كيفية منع الانفلونزا ونزلات البرد والسعال والتهابات الصدر في الحج بواسطة استخدام الكمامات (قناع الوجه). نحن ندعوك للانضمام لهذا البحث لأننا نأمل أن يتم منع انتشار عدوى الانفلونزا والتهابات الصدر الفيروسية بواسطة استخدام الكمامات (قناع الوجه) . وقد تمت دعوتك لأنك حاج/حاجة من أستراليا, السعودية أو قطر وعمرك فوق 18 عاما . ونهدف إلى مشاركة 7000 حاج معنا في هذه الدراسة .

إذا وافقت على المشاركة بهذه الدراسة، سنسألك عن بعض المعلومات المتعلقة بك وبصحتك . بعدها سنعطيك استبيانان (أحدهما لتعبئته يومياً أثناء الحج والآخر لحين رجعتك الى بلدك) . سيتم توزيع المشاركين عشوائيا إلى مجموعة من الخيام. مما يعني أن بعض الخيام سيتم تزويدها بالكمامات وليس الكل, بعد ذلك سنقوم بمتابعة صحتك.

وفي حالة إصابتك بنزلة البرد أو السعال أو الانفلونزا والتهابات الصدر فاطلب أحد أعضاء فريق البحث والذي بدوره سيزورك مرة واحدة في اليوم على الأقل لفحصك والعناية بك . سيأخذ الطبيب مسحة من أنفك (أو حلقك إذا كان أنسب لك) لمعرفة وتحديد الفيروس المسبب للالتهاب .

عند أخذ مسحة من الحلق أو الأنف قد تحس حينها بدغدغة أو برغبة في العطاس لكنها سرعان ما ستزول بعد ذلك .

سيتم صرف بعض الأدوية المسكنة للحرارة والآلام .

ليس عليك الانضمام إلى هذه الدراسة فالأمر متروك لك لتقرر . يمكنك أن توافق الآن ومن ثم تغير رأيك لاحقا بإخبارنا بأنك تريد التوقف . سواءاً لم ترد أن تكون في الدراسة أو إذا شاركت بالدراسة وغيرت رأيك لاحقاً وتوقفت, فلن يؤثر ذلك بشيءٍ عليك.

قبل أن تقول **نعم أو لا** لتكون في هذه الدراسة، فإننا سنرد على أية أسئلة لديك. إذا اشتركت بالدراسة، يمكنك طرح الأسئلة في أي وقت

إذا كان لديك أي أسئلة حول هذه الدراسة لا تتردد في الاتصال على:

- د. أسامة بارشيد ، موبايل: +966 503 362 068 أو إيميل : [barasheedoa@gmail.com](mailto:barasheedoa@gmail.com)
- د. هيثم البشير ، تلفون: 97455998276+ أو إيميل : [*helbashir@hmc.org.qa*](mailto:helbashir@hmc.org.qa)
- د. هارون رشيد، تلفون: +61 29845 1489 أو إيميل : [harunor.rashid@health.nsw.gov.au](mailto:harunor.rashid@health.nsw.gov.au)

إذا قمت بتسجيل اسمك أدناه، فهذا يعني أنك توافق على المشاركة في هذه الدراسة البحثية.

| توقيع المشارك البالغ | | | | |
| --- | --- | --- | --- | --- |
| أوافق طوعاً على الانضمام الى البحث المشروح في هذا النموذج | | | | |
| التاريخ |  | التوقيع |  | *الاسم الكامل للمشارك بالبحث* |
| الشخص الحاصل على الموافقة | | | | |
| أشهد أني:   - أنا (أو أحد أعضاء فريق البحث) قمنا بشرح البحث بشكل وافي للمشارك بالبحث - قمت شخصياً بتقييم فهم المشارك بالبحث والحصول على موافقته/ها الطوعية. | | | | |
| *التاريخ* |  | *التوقيع* |  | *الاسم الكامل الشخص الحاصل على الموافقة* |
