# Supplementary material for: Facemask against viral respiratory infections among Hajj pilgrims: A challenging cluster-randomized trial
Source: PLoS One. 2020 Oct 13;15(10):e0240287. doi: 10.1371/journal.pone.0240287 (PMC7553311; doi:10.1371/journal.pone.0240287)
Supplement: S2 Appendix — (DOC) [file pone.0240287.s003.doc]

**Cluster-randomised controlled trial to test the effectiveness of facemasks in preventing respiratory virus infection among Hajj pilgrims**

# Background

Viral respiratory tract infections (RTIs) are a major burden to public health, causing serious disease especially in the vulnerable population. These infections impose a substantial health burden in all countries. For instance, the World Health Organization (WHO) estimates that there are, annually, 1 billion cases of influenza during interpandemic years alone, with 3-5 million cases of severe illness and 300 000 – 500 000 deaths per year.^1^

Fear of the global spread of serious respiratory disease persists in light of past pandemics, such as the 2003 SARS coronavirus outbreak, 2009 H1N1 influenza virus, and the recent observation of the Middle East Respiratory Syndrome Coronavirus (MERS CoV), in Gulf countries and elsewhere, with reports of limited person to person transmission.^2^ Ever-increasing and fast international travel intensifies the risk as well as increases the likelihood of emergence of novel strains of viruses through genetic re-assortment thereby further compounding concern.^3^

Health authorities require cost-effective interventions that prevent, limit or slow the global transmission of respiratory diseases. Members of the public also require guidance to minimise the spread of infection.

Non-pharmaceutical interventions like personal protective measures such as facemasks and hand hygiene, and population interventions such as social distancing, quarantine and school closures may complement pharmaceutical measures like vaccination (eg, against influenza) and antiviral therapy (eg, anti-influenza drugs) in the prevention and control of viral RTIs. Facemasks could represent a simpler, cheaper alternative (or supplement) to the use of antivirals.

Results from studies examining the effectiveness of facemasks have been either conflicting or inconclusive.^4^ A randomised controlled trial (RCT) in a household setting found that adherence to facemask use decreased the risk of influenza-like illness (ILI), which was defined as fever and cough or sore throat .^5^ Two other RCTs found facemasks to be effective in reducing laboratory-confirmed influenza in households but only when implemented together with hand hygiene within 36 hours of a contact developing ILI.^6,7^ Aeillo and colleagues also found a similar result in the setting of university halls.^8^ However, three other studies in households did not find facemasks to be effective in reducing ILI.^9-11^ Surgical masks have been compared with N95 respirators in healthcare settings. Loeb and colleagues found that facemasks were non-inferior to N95, but MacIntyre and colleagues found N95 to be more protective than facemasks against clinical respiratory illness among health care workers.^12-14^

Meta-analysis of data from 6 trials shows that wearing facemasks is protective against ILI, but do not show that facemasks are protective against laboratory proven influenza (Figure 1).^15,7^ The major limitation of previous studies is believed to be the small sample size, and resulting lack of power to detect effects of facemasks on laboratory-proven influenza (and other respiratory viruses).^15^ The largest study so far was a cluster-randomised study in 509 households, with 2788 recruits, conducted by Larson and colleagues, but did not find an additional benefit of facemasks with hand hygiene over health education (regarding preventive measures) on overall rates of laboratory-proven viral infection, although there seemed to be a benefit on secondary transmission of respiratory infection; despite the size of this study, the authors pointed to concerns about under-powering and poor compliance with mask use.^10^ MacIntyre and colleagues’ individually randomised study with 1669 healthcare workers found no difference in rate of laboratory-proven respiratory pathogens between facemask and N95 arms.^14^ An observational study at Hajj showed that the use of a facemask by male pilgrims, but not a face-cover by females, was a significant protective factor against ARI while interestingly, a nested case-control study showed that intermittent facemask use was associated with a 2.7 fold greater risk of infection among health care workers at Hajj, suggesting perhaps that a facemask worn in the presence of an infected patient and contaminated with infectious material becomes the source of hand transmission (to the respiratory tract).^16,17^ Bin Reza *et al*. also consider that failure to reach a firm conclusion may have stemmed from inadequate sample size. ^4^ They therefore, recommended that sufficient power may be obtained by large multi-centred trials run for several years.^4^ Such a design at community settings will have considerable logistical and cost issues.


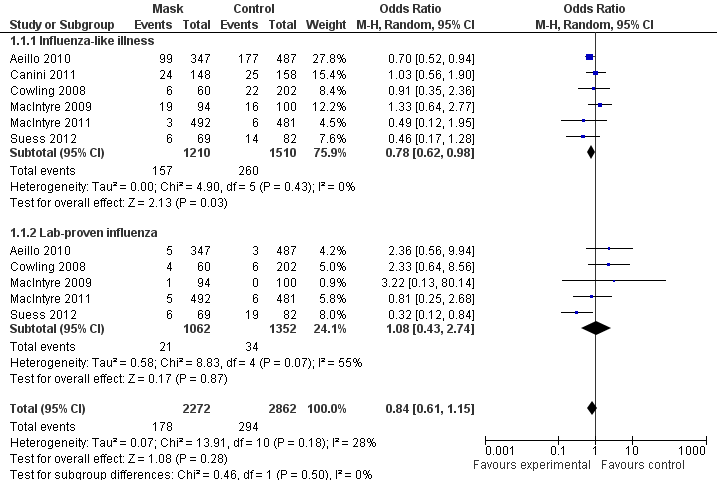


**Figure 1:** ***Comparison between mask and control groups in randomised trials*** (adapted from Rashid *et al*. with the inclusion of an additional study^7, 15^).

We have estimated that about 3500 subjects will be needed in each arm of a study to achieve sufficient power to detect a difference at the level of laboratory-confirmed respiratory viruses (please refer to sample size calculation section). Conducting such a trial at a mass gathering such as the Hajj, where annually about two and half million pilgrims gather from all corners of the world, over two years offers a viable alternative. Thus the logistical difficulties of recruiting a large number of subjects could be reduced. About 30,000 tents are used to house the pilgrims in Mina, a valley on the outskirts of Mecca. Pilgrims stay in these tents for at least 5 days. We would take the advantage of this ‘natural experiment’ to answer an important public health question whether facemasks are effective in preventing respiratory virus infection among Hajj pilgrims. This research is proposed at a time when the emergence of MERS CoV is threatening the world and therefore has an immediate public health implication.^2^

**Objectives**

*Primary aim*

1. To evaluate the efficacy of facemasks against laboratory-confirmed respiratory viral infections (including influenza, MERS CoV and other respiratory viruses) at Hajj 2013 and 2014.

*Secondary aims*

1. To investigate the transmission pattern of influenza virus at Hajj by studying the genetic relatedness between circulating influenza viruses using deep genomic sequencing.
2. To determine the self-reported influenza vaccine uptake and evaluate its effectiveness at Hajj using a test-negative case-control design.

**Research Plan**

**Design of the trial**

The key steps of the study are shown in Figure 2.

This study will be a large-scale cluster-randomised controlled trial to test the efficacy of facemasks in preventing acute respiratory viral infection including influenza and coronaviruses.

The trial will compare the ‘*use of (simple surgical) facemasks’* versus ‘*no use of facemasks’* among pilgrims during the Hajj week (13 to 20 October, 2013, and 2 to 9 October 2014). Randomisation to each arm will be in a 1:1 ratio and will be done according to tents. Computer-generated random numbers will be made by an offsite research coordinator who will not take part in recruitment or assessment of participants. The randomisation will be stratified by gender and country of residence to ensure a balanced and proportionate recruitment.

**Recruitment**

The study will aim to recruit pilgrims at the 2013 Hajj from Saudi Arabia, Qatar and Australia. Whereas in 2014, subjects will also be recruited from Indonesia, Malaysia, Turkey, Morocco, India, Pakistan and Bangladesh (Table 1). Before the commencement of Hajj, the tour groups will be provided with detailed information about the study and encouraged to participate in their respective countries of residence. At Hajj, trained study staff will approach tents for recruitment on the morning or afternoon of the first day of the pilgrim’s stay in Mina (corresponding to13^th^ October, 2013, and 2^nd^ October, 2014). The research team member will explain the study to the assembled pilgrims in each tent and invite them to join the study. If the pilgrims agree to participate, a consent form will be signed and a baseline questionnaire will be filled which will include collecting relevant demographics, vaccination history and clinical information such as respiratory symptoms with duration and pre-existing medical condition (Appendix 1). The participants will be informed whether their tent has been randomised to facemask wearing or no facemask wearing.

­

13^th^

October

Pilgrims settle into tents

Randomisation

Intervention tents Control tents

All fill baseline questionnaires

Given *Hajj diaries*

Receive masks and demonstration. Do not receive masks

Start wearing masks

14^th^ October

All record symptoms in Hajj diaries

Symptomatic pilgrims are swabbed

Continue mask wearing Do not wear masks

15^th^ October

16^th^ October

Study staff collect *Hajj diaries* in the evening

17^th^ October

18^th^ October

Evening

19^th^ October

Evening

Pilgrims leave Mina and start keeping *post-Hajj* diaries

20^th^ October

No need to keep *post-Hajj* diaries

Mask wearing is no more required No need to avoid mask wearing

Nasal swabs get transported to Sydney

Pilgrims send *post-Hajj* diaries to research physicians

**Figure 2: Key steps of the study at the Hajj 2013**

**Inclusion criteria**
Pilgrims from participating countries staying in their respective tents
Age ≥18 years.
Informed consent has been agreed and signed.

**Exclusion criteria**
Children aged less than 18 years.
Participation in another clinical trial investigating a medical intervention that may interfere with study outcome measures like proven viral RTI.
Known contraindication to mask use (eg, allergy to surgical mask materials).

Refused to sign a consent form.

**Data collection**

For the tents assigned to mask use, 25 masks will be provided to each participant, one to be worn continuously (24/7), and then replaced every few hours (sooner if wet), for the 5 days at Mina.

The mask for this study is ‘3M™ Standard Tie-On Surgical Mask, Cat No: 1816’. Masks may be removed for eating, praying, washing, brushing teeth, or if the pilgrim experiences discomfort. Oral and written information about the instructions for correct mask wearing and disposal will be provided to the mask group (Appendix 2). Study staff will practically demonstrate the correct method of how to wear masks and help pilgrims to put on their mask for the first time. Masks can be changed every 4- 6 hours or more frequently if the masks become damp or damaged. Handouts will be distributed to all pilgrims at ‘mask tents’ to encourage correct mask wearing and explain the correct disposal of used masks. Small plastic bags will be provided for mask disposal and subjects will be instructed to discard the plastic bag containing used masks into waste bins provided for subsequent sanitary disposal. A subset of disused masks can be saved for further testing by multiplex viral PCR. A research team member will be available each morning and evening to distribute additional facemasks should pilgrims request extra, and to document any reported ILI symptoms development.

On recruitment, study staff will record each tent number, the number of people recruited and the total number of people in the tent. All pilgrims will be given a *Hajj health* diary (Appendix 3) for 5 nights at Mina (corresponding to 13-17 October, 2013, and 2-6 October, 2014). Each diary will have a unique barcode identifying the diaries with a specific study participant. The diaries will contain questions on demographics, medical conditions, and influenza vaccination history (Appendices 3 and 4). Each participant will provide information about the presence or absence of respiratory symptoms and fever in the diary every day whenever it is most convenient (eg after their evening meal). Pilgrims will also record in their diaries the amount of time they have worn the mask, the number of masks used per day and whether they were wearing one in their sleep.

No mask will be provided to anyone in the ‘no mask’ tents (although pilgrims may use their own supply of masks) but they will be enrolled in the study, informed consent will be obtained and the baseline questionnaire completed, then they will be given diaries to record their ILI symptoms and encouraged to record relevant information.

The *Hajj health* diaries will be collected on the 4^th^ day evening from both ‘mask’ and ‘no mask’ groups when *post-Hajj health* diaries will be distributed to these pilgrims with self-addressed prepaid envelopes. Pilgrims will be instructed to post these diaries to the researchers. Pilgrims whose *Hajj health* diaries were not collected on the 4^th^ day will be instructed to send those along with *post-Hajj health* dairies (Appendix 4) in the same envelope.

**Specimen collection and testing**

In both groups of tents the study team members will actively search for pilgrims suffering from ILI (defined as subjective or measured fever and at least one respiratory symptom such as cough, sore throat and rhinorrhea) at least once every day. A study team member will collect nasopharyngeal (NP) swab (or throat swab if an NP swab is not doable) for molecular diagnostic testing for respiratory viruses. As a part of the routine care pilgrims in both tent groups will be supplied with generic medications (such as acetaminophen and ibuprofen) for fever or aches in addition to antiviral drugs for confirmed influenza patients.

The swabs will be stored within 2-3 hours at subzero temperature for later molecular diagnostic testing at the Centre for Infectious Diseases and Microbiology Laboratory Services (CIDMLS), Westmead Hospital, NSW, Australia. Pilgrims will provide their email addresses, phone numbers and mail addresses if they wish to receive the results of the laboratory test. Multiplex reverse transcriptase polymerase chain reaction (RT-PCR) for influenza A and B, MRES CoV and other corona viruses, parainfluenza viruses (types 1,2, 3 and 4), RSV A and B, adenoviruses, human metapneumovirus (hMPV), and picorna viruses will be performed. Nasal swabs will be taken for reverse transcriptase polymerase chain reaction (RT-PCR) analysis of respiratory virus infection. The swab used will be Copan nylon flocked dry nasal swab. The study staff who will take the swab will all have been trained to achieve an appropriate sample.

To determine the transmission patterns of influenza viruses among Hajj pilgrims, complete genome sequence of influenza virus will be obtained using a next generation sequencing protocol – using the Illumina MiSeq sequencer available at Westmead Hospital – and which will generate multiple reads per host (~1000X coverage per host; that is, ‘deep’ sequencing). All sequence data generated will be assembled and aligned using the Geneious (http://www.geneious.com) and VICUNA packages,^18^ with downstream phylogenetic (and other evolutionary) analysis undertaken using the Geneious, Seminator,^19^ and PhyML packages.^20^ With these data in hand we will be able to determine; (i) whether the study participants were infected prior or during the Hajj, (ii) whether there was direct viral transmission among the study participants (such that they harbor both ‘majority’ and ‘minority’ genetic variants), and (iii) if direct transmission is established whether this occurs more frequently in the mask versus control groups.

Respiratory samples will not stored or banked for more than it is necessary (eg, to revalidate any result). Any leftover sample will be disposed according to the standard operating procedure of the CIDMLS.

**Follow up**

All participants will be followed up (e.g. by telephone, mails, emails) by study physicians in their country of residence to collect their *post-Hajj health* diaries (as well as *Hajj health* dairies if not collected earlier). Having obtained prior consent from the participants to do so, their GPs/family physicians (if available) may be contacted to validate any clinical detail, especially to verify vaccination histories.

**Data analysis**

Data available from questionnaires, diaries and laboratory tests will be analysed anonymously to examine whether mask use makes a significant difference in reducing the frequency of laboratory-confirmed respiratory virus infection (including influenza, corona virus or other respiratory viruses). The primary endpoints (effectiveness and efficacy of facemasks against ILI and laboratory-confirmed viruses respectively) will be analysed by intention to treat.

The self-reported uptake rates of influenza vaccination will be determined and vaccine effectiveness will be estimated based on the case-negative case-control methodology.

The results from the genomic sequencing of influenza virus will assist us understanding the genetic relatedness of circulating influenza strains at Hajj and the transmission pattern of influenza among pilgrims.

**Sample size calculation**

Assuming that the prevalence of symptomatic RTIs is 30% in the controls and the prevalence of laboratory-proven respiratory viral infection in controls is approximately 12% the intervention could be considered clinically worthwhile if it can reduce the prevalence of syndromic or proven viral RTI by 50%.^21,22^

Assuming a moderate intra-cluster correlation of 10% and a mean of 75 participants per cluster (tent), and inflating the sample by a factor of 8.4 to account for clustering, the sample size required for a cRCT to detect a reduction from 12% to 6% with 80% power at 5% significance is 2976 per arm. An additional inflation factor of 1.18 will allow for up to 15% loss to follow-up or incomplete outcome data. This results in a sample size of approximately 3500 participants per treatment making a total of 7000.

For the first primary outcome (clinical/syndromic RTIs) a smaller sample would be sufficient to answer a more generic question, namely prevention of symptomatic RTI. The sample size required for detecting a reduction from 30% prevalence of RTIs to 15% with 80% power at 5% significance and considering for clustering and loss to follow-up is about 1170 participants per treatment making a total of 2340.

| **Countries** | **Total number of pilgrims** | **Number of recruits** | |
| --- | --- | --- | --- |
|  |  | **2013** | **2014** |
| Saudi Arabia | 1,000,000 | 2,200 | 2,200 |
| Indonesia | 200,000 |  | 500 |
| India | 170,000 |  | 450 |
| Pakistan | 150,000 |  | 350 |
| Turkey | 120,000 |  | 300 |
| Bangladesh | 65,000 |  | 200 |
| Morocco | 32,000 |  | 100 |
| Malaysia | 28,000 |  | 100 |
| Australia | 5,000 | 200 | 200 |
| Qatar | 2,000 | 100 | 100 |
| Total | 1,770,000 | 2500 | 4500 |

**Table 1: Proposed number of pilgrims from each participating country**

To achieve the full sample size we aim to conduct the study over two years (2013 and 2014). About 2500 pilgrims (or at least 2340) will be recruited in 2013 which will give an answer for the RTI outcome and could build on that sample for the second outcome (proven infection) which ultimately will be completed by recruiting another 4500 pilgrims in 2014. Proportionate (or balanced) number of pilgrims will be recruited from each participating country according to the number of pilgrims attending Hajj from these countries (see Table 1).

**Ethics implication**

We got ethics approval to conduct a pilot study involving Australian pilgrims until December 2013 (reference: 11/SCHN/162). We also obtained a Saudi Arabian ethics approval for the pilot trial (reference: KACST: H-01-R-012), and a fresh application is being made for this main trial. Ethics approval is being sought from the Joint Institutional Review Board (J-IRB) of Hamad Medical Corporation - Weill Cornell Medical College in Qatar. The study coordinator will provide a modified ethics committee submission for collaborators and assist them to apply for ethical approval from the relevant Ethics Committees. Only participants who provide written informed consent to take part will be included in the study. Participants will be told that taking part in this study would be voluntary and confidential. All participants will be assigned a study identification (ID) number. Data will be collected and stored under this number only, so that all data are stored anonymously by study ID number only. Hard copies of the questionnaires will be stored securely in a locked filing cabinet in locked offices and electronic data will be stored in password-protected computer files. Only members of the research team will have access to the data. The hard copies will be retained for at least fifteen years (or more, if the data management protocol of the participating country requires so) and will be disposed according to standard data management guidelines.

**References**

1. World Health Organization: Global influenza programme: pandemic influenza preparedness and response, *WHO guidance document* 2009.

2. de Groot RJ, Baker SC, Baric RS, *et al*. Middle East Respiratory Syndrome Coronavirus (MERS-CoV); Announcement of the Coronavirus Study Group. *J Virol* 2013 (in press).

3. Balkhy H, Al-Hajjar S. Avian influenza: are our feathers ruffled? *Ann Saudi Med* 2006; **26**:175-82.

4. BinReza F, Chavarraias VL, Nicoll A, Chamberland ME. The use of masks and respirators to prevent transmission of influenza: a systematic review of the scientific evidence. *Influenza and Other Respiratory Viruses* 2011; **6**:257-67.

5. MacIntyre RC, Cauchemez S, Dwyer DE, *et al*. Face mask use and control of respiratory virus transmission in households. *Emerging Infectious Diseases* 2009; **15**:233-41.

6. Cowling BJ, Chan KH, Fang VJ, *et al*. Facemasks and hand hygeine to prevent influenza transmission in households, a cluster randomized trial. *Annals of Internal Medicine* 2009; **151**:437-46.

7. Suess T, Remschmidt C, Schink SB, *et al*. The role of facemasks and hand hygiene in the prevention of influenza transmission in households: results from a cluster randomized trial; Berlin, Germany, 2009-2011. *BMC Infectious Diseases* 2012; **12**:26.

8. [Aiello AE](http://www.ncbi.nlm.nih.gov/pubmed?term=Aiello AE%5BAuthor%5D&cauthor=true&cauthor_uid=20088690), [Murray GF](http://www.ncbi.nlm.nih.gov/pubmed?term=Murray GF%5BAuthor%5D&cauthor=true&cauthor_uid=20088690), [Perez V](http://www.ncbi.nlm.nih.gov/pubmed?term=Perez V%5BAuthor%5D&cauthor=true&cauthor_uid=20088690), [*et*](http://www.ncbi.nlm.nih.gov/pubmed?term=Coulborn RM%5BAuthor%5D&cauthor=true&cauthor_uid=20088690) *al*. Mask use, hand hygiene, and seasonal influenza-like illness among young adults: a randomized intervention trial. [*Journal Infectious Diseases*](http://www.ncbi.nlm.nih.gov/pubmed/?term=Mask+Use%2C+Hand+Hygiene%2C+and+Seasonal+Influenza-+Like+Illness+among+Young+Adults%3A+A+Randomized+Intervention+Trial) 2010; **201**:491-8.

9. [Canini L](http://www.ncbi.nlm.nih.gov/pubmed?term=Canini L%5BAuthor%5D&cauthor=true&cauthor_uid=21103330), [Andréoletti L](http://www.ncbi.nlm.nih.gov/pubmed?term=Andréoletti L%5BAuthor%5D&cauthor=true&cauthor_uid=21103330), [Ferrari P](http://www.ncbi.nlm.nih.gov/pubmed?term=Ferrari P%5BAuthor%5D&cauthor=true&cauthor_uid=21103330), [*et*](http://www.ncbi.nlm.nih.gov/pubmed?term=D'Angelo R%5BAuthor%5D&cauthor=true&cauthor_uid=21103330) *al*. Surgical Mask to prevent influenza transmission in households: a cluster randomised trial. *PLoS One* 2010; **5**(11):e13998.

10. [Larson EL](http://www.ncbi.nlm.nih.gov/pubmed?term=Larson EL%5BAuthor%5D&cauthor=true&cauthor_uid=20297744), [Ferng YH](http://www.ncbi.nlm.nih.gov/pubmed?term=Ferng YH%5BAuthor%5D&cauthor=true&cauthor_uid=20297744), [Wong-McLoughlin J](http://www.ncbi.nlm.nih.gov/pubmed?term=Wong-McLoughlin J%5BAuthor%5D&cauthor=true&cauthor_uid=20297744), [Wang S](http://www.ncbi.nlm.nih.gov/pubmed?term=Wang S%5BAuthor%5D&cauthor=true&cauthor_uid=20297744), [Haber M](http://www.ncbi.nlm.nih.gov/pubmed?term=Haber M%5BAuthor%5D&cauthor=true&cauthor_uid=20297744), [Morse SS](http://www.ncbi.nlm.nih.gov/pubmed?term=Morse SS%5BAuthor%5D&cauthor=true&cauthor_uid=20297744). Impact of non-pharmaceutical interventions in URIs and influenza in crowded, urban households. *Public Health Reports* 2010; **125**:178-91.

11. [Simmerman JM](http://www.ncbi.nlm.nih.gov/pubmed?term=Simmerman JM%5BAuthor%5D&cauthor=true&cauthor_uid=21651736), [Suntarattiwong P](http://www.ncbi.nlm.nih.gov/pubmed?term=Suntarattiwong P%5BAuthor%5D&cauthor=true&cauthor_uid=21651736), [Levy J](http://www.ncbi.nlm.nih.gov/pubmed?term=Levy J%5BAuthor%5D&cauthor=true&cauthor_uid=21651736), [*et*](http://www.ncbi.nlm.nih.gov/pubmed?term=Jarman RG%5BAuthor%5D&cauthor=true&cauthor_uid=21651736) *al*. Findings from a household randomized controlled trial of hand washing and face masks to reduce influenza transmission in Bangkok, Thailand. [*Influenza Other Respiratory Viruses*](http://www.ncbi.nlm.nih.gov/pubmed/?term=Findings+from+a+household+randomized+controlled+trial+of+hand+washing+and+face+masks+to+reduce+influenza+transmission+in+Bangkok%2C+Thailand) 2011; **5**:256-67.

12. Loeb M, Dafoe N, Mahony J, *et al*. Surgical mask vs N95 respirator for preventing influenza among health care workers: a randomized trial. *JAMA* 2009; **302**:1865-71.

13. [MacIntyre CR](http://www.ncbi.nlm.nih.gov/pubmed?term=MacIntyre CR%5BAuthor%5D&cauthor=true&cauthor_uid=21477136), [Wang Q](http://www.ncbi.nlm.nih.gov/pubmed?term=Wang Q%5BAuthor%5D&cauthor=true&cauthor_uid=21477136), [Cauchemez S](http://www.ncbi.nlm.nih.gov/pubmed?term=Cauchemez S%5BAuthor%5D&cauthor=true&cauthor_uid=21477136), [*et*](http://www.ncbi.nlm.nih.gov/pubmed?term=Seale H%5BAuthor%5D&cauthor=true&cauthor_uid=21477136) *al*. A cluster randomized clinical trial comparing fit-tested and non-fit-tested N95 respirators to medical masks to prevent respiratory virus infection in health care workers. [*Influenza and Other Respiratory Viruses*](http://www.ncbi.nlm.nih.gov/pubmed/?term=A+cluster+randomized+clinical+trial+comparing+fit-tested+and+non-fit-tested+N95+respirators+to+medical+masks+to+prevent+respiratory+virus+infection+in+health+care+workers) 2011; **5**:170-9.

14. [Macintyre CR](http://www.ncbi.nlm.nih.gov/pubmed?term=Macintyre CR%5BAuthor%5D&cauthor=true&cauthor_uid=23413265), [Wang Q](http://www.ncbi.nlm.nih.gov/pubmed?term=Wang Q%5BAuthor%5D&cauthor=true&cauthor_uid=23413265), [Seale H](http://www.ncbi.nlm.nih.gov/pubmed?term=Seale H%5BAuthor%5D&cauthor=true&cauthor_uid=23413265), [*et*](http://www.ncbi.nlm.nih.gov/pubmed?term=Yang P%5BAuthor%5D&cauthor=true&cauthor_uid=23413265) *al*. A randomised clinical trial of three options for N95 respirators and medical masks in health workers. [*Am J Respir Crit Care Med*](http://www.ncbi.nlm.nih.gov/pubmed/?term=A+randomised+clinical+trial+of+three+options+for+N95+respirators+and+medical+masks+in+health+workers) 2013 (in press).

15. [Rashid H](http://www.ncbi.nlm.nih.gov/pubmed?term=Rashid H%5BAuthor%5D&cauthor=true&cauthor_uid=22187415), [Booy R](http://www.ncbi.nlm.nih.gov/pubmed?term=Booy R%5BAuthor%5D&cauthor=true&cauthor_uid=22187415), [Heron L](http://www.ncbi.nlm.nih.gov/pubmed?term=Heron L%5BAuthor%5D&cauthor=true&cauthor_uid=22187415), [et](http://www.ncbi.nlm.nih.gov/pubmed?term=Memish ZA%5BAuthor%5D&cauthor=true&cauthor_uid=22187415) al. Unmasking masks in Makkah: preventing influenza at Hajj. [*Clin Infect Dis*](http://www.ncbi.nlm.nih.gov/pubmed/?term=Unmasking+masks+at+makkah) 2012; **54**:151-3.

16. Choudhry AJ, Al-Mudaimegh KS, Turkistani AM, Al-Hamdan NA. Hajj-associated acute respiratory infection among hajjis from Riyadh. *East Mediterr Health J* 2006; **12**:300-9.

17. [Al-Asmary S](http://www.ncbi.nlm.nih.gov/pubmed?term=Al-Asmary S%5BAuthor%5D&cauthor=true&cauthor_uid=16905350), [Al-Shehri AS](http://www.ncbi.nlm.nih.gov/pubmed?term=Al-Shehri AS%5BAuthor%5D&cauthor=true&cauthor_uid=16905350), [Abou-Zeid A](http://www.ncbi.nlm.nih.gov/pubmed?term=Abou-Zeid A%5BAuthor%5D&cauthor=true&cauthor_uid=16905350), [Abdel-Fattah M](http://www.ncbi.nlm.nih.gov/pubmed?term=Abdel-Fattah M%5BAuthor%5D&cauthor=true&cauthor_uid=16905350), [Hifnawy T](http://www.ncbi.nlm.nih.gov/pubmed?term=Hifnawy T%5BAuthor%5D&cauthor=true&cauthor_uid=16905350), [El-Said T](http://www.ncbi.nlm.nih.gov/pubmed?term=El-Said T%5BAuthor%5D&cauthor=true&cauthor_uid=16905350). Acute respiratory tract infections among Hajj medical mission personnel, Saudi Arabia. [*Int J Infect Dis*](http://www.ncbi.nlm.nih.gov/pubmed/?term=Acute+respiratory+tract+infections+among+Hajj+medical+mission+personnel%2C+Saudi+Arabia) 2007; **11**:268-72.

18. Yang X, Charlebois P, Gnerre S, *et al*. De novo assembly of highly diverse viral populations. *BMC Genomics* 2012;**13**:475.

19. Archer J, Baillie G, Watson SJ, Kellam P, Rambaut A, Robertson DL. Analysis of high-depth sequence data for studying viral diversity: a comparison of next generation sequencing platforms using Segminator II. *BMC Bioinformatics* 2012;**13**:47.

20. Guindon S, Dufayard JF, Lefort V, Anisimova M, Hordijk W, Gascuel O. New algorithms and methods to estimate maximum-likelihood phylogenies: assessing the performance of PhyML 3.0. *Syst Biol* 2010; **59**:307-21.

21. Al-Tawfiq JA, Zumla A, Memish ZA. Respiratory tract infections during the annual Hajj: potential risks and mitigation strategies. *Curr Opin Pulm Med* 2013; **19**:192-7.

22. [Memish ZA](http://www.ncbi.nlm.nih.gov/pubmed?term=Memish ZA%5BAuthor%5D&cauthor=true&cauthor_uid=22221807), [Assiri AM](http://www.ncbi.nlm.nih.gov/pubmed?term=Assiri AM%5BAuthor%5D&cauthor=true&cauthor_uid=22221807), [Hussain R](http://www.ncbi.nlm.nih.gov/pubmed?term=Hussain R%5BAuthor%5D&cauthor=true&cauthor_uid=22221807), [Alomar I](http://www.ncbi.nlm.nih.gov/pubmed?term=Alomar I%5BAuthor%5D&cauthor=true&cauthor_uid=22221807), [Stephens G](http://www.ncbi.nlm.nih.gov/pubmed?term=Stephens G%5BAuthor%5D&cauthor=true&cauthor_uid=22221807). Detection of respiratory viruses among pilgrims in Saudi Arabia during the time of a declared influenza A(H1N1) pandemic. [*J Travel Med*](http://www.ncbi.nlm.nih.gov/pubmed/?term=detection+of+respiratory+viruses+among+pilgrims+in+saudi) 2012; **19**:15-21.

**All appendices mentioned here are in ‘Supplementary Appendix’ (S1 Appendix).**

| **Document** | **Original name** | **Page number** |
| --- | --- | --- |
| Appendix 1 | Baseline Questionnaire English | 7 |
| Appendix 2 | Instructions to Use Facemasks English | 10 |
| Appendix 3 | Hajj Diary English. | 11 |
| Appendix 4 | Post-Hajj Diary English | 20 |
